# COVID-19 mRNA-1273 vaccination induced mast cell activation with strongly elevated Th_2_ cytokines in a systemic mastocytosis patient

**DOI:** 10.1007/s00011-025-02032-5

**Published:** 2025-04-29

**Authors:** Matthias Weiss-Tessbach, Teresa Haider, Aoife Gowran, Lorenz Schubert, Jakob Mühlbacher, Jelena Brankovic, Markus Wahrmann, Bernd Jilma, Thomas Boehm

**Affiliations:** 1 Department of Clinical Pharmacology, Medical University Vienna, Waehringer Guertel 18-20, Vienna, 1090 Austria; 2 Division of Neuropathology and Neurochemistry, Department of Neurology, Medical University Vienna, Vienna, Austria; 3 Department of Medicine I, Division of Infectious Diseases and Tropical Medicine, Medical University Vienna, Vienna, Austria; 4 Department of Surgery, Division of Visceral Surgery, Medical University Vienna, Vienna, Austria; 5 Division of Nephrology and Dialysis, Department of Medicine III, Medical University Vienna, Vienna, Austria

**Keywords:** Systemic mastocytosis, mRNA-1273 vaccination, Th_2_-biased cytokines, Tocilizumab, Histamine, SARS-Cov-2

## Abstract

**Objective and design:**

SARS-CoV-2 vaccines are recommended for mastocytosis patients. We describe clinical symptoms, chemokine, cytokine, metabolomic and lipidomic derangements in a systemic mastocytosis patient following mRNA-1273 booster vaccination.

**Methods:**

Twenty-eight chemokines and cytokines, 41 amino acids and 16 lipid classes were quantified with state-of-the-art methods.

**Results:**

Mast cell activation (MCA) symptoms started 24 h after the mRNA-1273 booster vaccination with significant metabolic, lipidomic and cytokine derangements. Histamine concentrations peaked at life-threatening 18 ng/ml concomitant with high tryptase. Peak plasma IL-1Ra, IL-5, IL-6, IL-10, IL-11, CXCL10 and GM-CSF concentrations were elevated 54-, 4.9-, 85-, 54-, 6.1-, 19- and 6.4-fold respectively. Tocilizumab, an IL-6 receptor antagonist, was administered 6 h after admission, because of the highly elevated IL-6 concentrations. More than one year later IL-6 was highly elevated during another MCA attack likely caused by a PCR-proven SARS-CoV-2 infection and tocilizumab was again used. Clinical symptoms improved during the following 12 h similar to the vaccine booster MCA attack.

**Conclusions:**

A mRNA-1273 first booster vaccination likely caused a delayed severe MCA attack with highly elevated Th_2_-biased cytokines with metabolic and lipidomic derangements. Administration of an IL-6 receptor blocker during both MCA attacks might have shortened the duration of clinical symptoms.

**Supplementary Information:**

The online version contains supplementary material available at 10.1007/s00011-025-02032-5.

## Introduction

Soon after the first mRNA vaccines were authorized for the prevention of coronavirus disease 2019 (COVID-19), reports of anaphylactic reactions were published [1]. Although the published incidence rate is relatively low at 12.8 cases per million doses, the widespread use of SARS-CoV-2 vaccinations and potentially lethal consequences raised public concern. The use of a new technology with a shortened clinical development path was not helpful in the amelioration of these negative perceptions [2]. Definitive trigger molecules for the anaphylactic reactions after vaccination have not been identified. Possible culprits include mRNA-based activation of pathogen-associated molecular pattern receptors, activation of the contact system, complement-mediated reaction to lipid nanoparticles, immune system activation via pre-existing anti-polyethylene glycol (PEG) antibodies and direct activation of mast cells by lipid nanoparticles [3]. 

Primary mast cell activation syndrome (MCAS) or mastocytosis is characterized by an abnormal expansion of mast cells in various organs, which renders systemic mast cell activation (MCA) lifethreatening [4]. Mast cell activation can be induced by diverse stimuli like allergenic IgE/FcεR1 interactions, viral and bacterial products, cytokines, chemokines, cold, heat, food, exercise or medications with many events still induced by an unknown trigger [5].

Because of the frequently unknown and unpredictable trigger molecules of MCA [6], the newly developed mRNA vaccines were carefully and closely evaluated in individuals with mastocytosis. Following a benefit-risk assessment the European and American mastocytosis societies recommended mRNA-based SARS-CoV-2 vaccinations for all mastocytosis patients over 16 years of age with certain precautions such as prolonged supervision after vaccination and pre-medication with histamine 1 and 2 receptor antagonists [7]. Recent retrospective data of 323 patients with clonal (*n* = 276), non-clonal (*n* = 18) MCA disorders and α-tryptasemia (*n* = 29) support this recommendation by detecting only mild adverse reactions after vaccination [8]. Only the first 2 h after vaccination were considered “related to vaccine”. In a small prospective study 26 patients with clonal mast cell disorders had no MCA symptoms following vaccination [9]. These subjects were premedicated with both histamine 1 and 2 receptor antagonists and a leukotriene receptor antagonist. Lazarinis and co-authors reported that of 73 fully vaccinated mastocytosis patients only 2.7% reacted with mild immediate symptoms [10]. However, they included only the first 45 min in their evaluation of vaccination-related symptoms. Nevertheless, two cases of anaphylaxis Grade 3 (flushing, pruritus, presyncope and vocal hoarseness) and Grade 4 (urticaria, flushing, shortness of breath, palpitations and presyncope) have been described among 130 adult mastocytosis patients [11]. These events happened within 30 min.

Cytokine storms or cytokine release syndromes can be induced by malignant diseases, the application of immunotherapeutic drugs, autoimmune conditions or pathogens [12]. A cytokine storm might be defined by excessive circulating cytokine and chemokine concentrations, acute systemic inflammatory symptoms and secondary organ dysfunction [13]. A Th_1_biased cytokine release syndrome occurred in a cancer patient 5 days after mRNA SARS-CoV-2 vaccination [14]. A Th_2_-biased cytokine storm with more than 40-fold elevated circulating levels of interleukin-1Ra (IL-1Ra), IL-5, IL-6 and IL-10 was recently published in a systemic mastocytosis patient during prolonged MCA with no identified pathogen and with no clinical symptoms of an acute infection except elevated temperature [15].

In this report, we describe in a systemic mastocytosis patient first clinical symptoms and chemokine, cytokine, amino acid and lipid derangements during a severe MCA event likely caused by a SARS-CoV-2 mRNA-1273 first booster vaccination and second the clinical course of a MCA event 442 days later likely triggered by a PCR-proven SARS-CoV-2 infection. Because of the highly elevated IL-6 concentrations the IL-6 receptor blocking antibody tocilizumab was administered during both events approximately 6 h after hospital admission.

## Materials and methods

### Mast cell activation (MCA) after mRNA vaccination

A 43-year-old Caucasian with indolent systemic mastocytosis driven by a c-Kit mutation (D816V) diagnosed in 2008 was admitted to the hospital with symptoms of an acute MCA event after his first SARS-CoV-2 booster vaccination (100 µg mRNA-1273, Moderna Inc., USA), from now on referred to as the “MCA-vaccination” or first booster vaccination event. The patient reported no adverse effects after his first SARS-CoV-2 vaccination with the same vaccine mRNA-1273. More details about this patient including derangements of the lipidome, metabolome and chemo-/cytokinome during extended severe MCA events without identifiable cause have been published [15, 16]. 

**Fig. 1:**
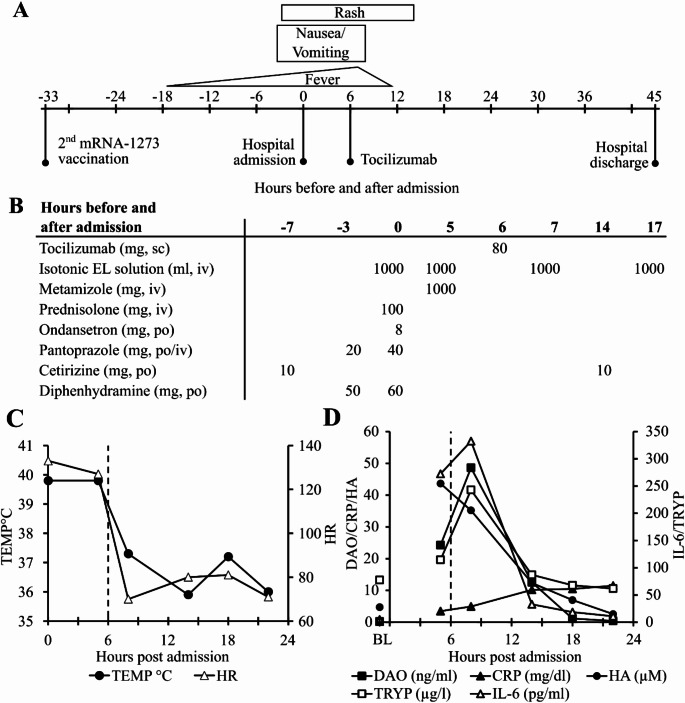
Time course of symptoms, administered substances and selected clinical and laboratory parameters during mast cell activation triggered by SARS-CoV-2 mRNA-1273 booster vaccination. **A** Clinical symptoms and time course of MCA-vaccination event with 0 h corresponding to hospital admission. **B** Medications administered before and during the MCA-vaccination event. **C** Time course of axillary temperature (TEMP) in degrees Celsius and heart rate (HR) per minute during the first 24 h after admission. **D** Time course of diamine oxidase (DAO, ng/ml), tryptase (TRYP, µg/l), C-reactive protein (CRP, mg/dl), histamine (HA, µM) and interleukin-6 (IL-6, pg/ml) concentrations. Baseline (BL) samples were obtained 2 weeks after the MC activation event. In Panels C and D the x-axis shows hours after hospital admission. Dashed vertical lines indicate tocilizumab administration 6 h after hospital admission. sc = subcutaneous, iv = intravenous, po = per os, EL solution = electrolyte solution, CRP = C-reactive protein, HA = histamine (semiquantitative), TRYP = tryptase, IL-6 = interleukin-6

Sixteen hours after the first booster mRNA-1273 vaccination the patient experienced fever, nausea combined with multiple episodes of vomiting. One hour before admission the patient reported the appearance of a whole-body rash. Ten mg cetirizine, 50 mg diphenhydramine and 20 mg pantoprazole were self-administered at home. Due to worsening symptoms the patient went to a primary care hospital at 18:00, where he intravenously received 100 mg prednisolone, 8 mg ondansetron, 40 mg pantoprazole and 60 mg diphenhydramine (Fig. 1B). After three hours at the primary care facility, the patient was transferred to the Department of Internal Medicine I of the Medical University of Vienna (MUV), where he received intravenous fluids and 1000 mg metamizole. Based on previous IL-6 cytokine data [15] and the highly elevated plasma IL-6 levels at admission, the patient received 80 mg of the anti-IL-6 receptor antibody tocilizumab 6 h after admission. The symptoms improved rapidly in the following hours. The patient was discharged 45 h after admission with no apparent symptoms.

Fourteen independent plasma samples at 5, 8, 14, 18, 22, 38, 45 h after hospitalization and two plasma baseline samples 2 weeks after dismissal were collected. At baseline the patient had no evidence of MCA, infection or any other pathological findings. Two independent EDTA blood samples were collected at each time point. One contained 20 µM diminazene aceturate (D7770, Sigma-Aldrich, Austria), a potent and specific diamine oxidase (DAO) inhibitor [17]. The DAO inhibitor was added to empty EDTA collection tubes without disruption of the vacuum before blood draw. This step was taken to inhibit histamine degradation by potentially released DAO from extracellular gastrointestinal storage sites. The patient provided informed consent for all therapeutic and investigative procedures. The study involving patients with mastocytosis received approval from the local ethics committee of the MUV under ethics committee number EC:1012/2013. During the events the patient provided informed consent and was included in the non-interventional anaphylaxis clinical study EC:1018/2015.

### Mast cell activation during SARS-CoV-2 infection

Four hundred and forty-two days after the SARS-CoV-2 booster vaccination the patient developed a sore throat and limb pain. A PCR test for the SARS-CoV-2 Omicron variant BA.4/BA.5 with mutation pattern S371F S373P, L452R, E484A and F486V was positive. Two days after the initial symptoms the patient started vomiting, experienced a rash and developed fever. Because symptoms worsened, the patient was admitted to the Emergency Department at the MUV. This event is from now on referred to as the “MCA-infection” event.

Three hours after admission the patient received 80 mg of the anti-IL-6 receptor antibody tocilizumab, because plasma IL-6 concentrations were again highly elevated. The rash gradually improved and the fever receded. The patient was discharged 24 h after admission.

### Measurements of other laboratory parameters

Laboratory parameters were measured in the Department of Laboratory Medicine of the MUV using accredited standardized methods unless stated otherwise. Diamine oxidase antigen concentrations were measured as described [18]. The activity of DAO was quantified using a recently published hybrid assay [19]. Briefly, plasma samples with and without the reversible DAO inhibitor diminazene aceturate from all time points were added to microtiter plates coated with a mouse monoclonal anti-human DAO antibody. During the following washing steps the potent DAO inhibitor diminazene aceturate is rapidly removed from the active center of the enzyme. The enzymatic activity was determined using cadaverine as DAO substrate with horseradish peroxidase (HRP) hydrogen peroxide (H_2_O_2_) coupling [19]. The detection limit of this assay is below 2 ng/ml DAO enzyme [20]. 

### Chemokine and cytokine measurements

Plasma samples with and without 20 µM diminazene aceturate were thawed for the first time after storage at − 80 °C and measured using a custom-built Luminex 28-plex kit (R&D Systems, Minneapolis, MN, USA) including Interleukin (IL)-1α, IL-1β, IL-1 receptor antagonist (IL-1Ra), IL-2, IL-3, IL-4, IL-5, IL-6, IL-8, IL-10, IL-11, IL-13, IL-15, IL-33, CC chemokine ligand (CCL)-2, CCL-3, CCL-4, CCL-11, CCL-20, tumor necrosis factorα (TNF-α), interferonα (IFN-α), IFN-β, IFN-γ, granulocyte macrophage-colony stimulating factor (GM-CSF), basic fibroblast growth factor (bFGF), granulocyte colony stimulating factor (G-CSF), platelet-derived growth factor (PDGF)-AA, and the C-X-C motif chemokine ligand CXCL-10 (previously IP-10). Standards were measured in duplicate and samples in triplicate on the same microtiter plate. A Luminex 200 analyzer was used to obtain fluorescence data. The xPONENT^®^ Version 3.1 software was used to convert fluorescence data into concentrations.

### Lipidome and metabolome analysis

Lipidomic and metabolomic analysis was performed in the Metabolomics Facility at the Center for Molecular Medicine (CeMM, Vienna, Austria). Detailed descriptions on both analytical methods are provided in the supplementary material. Functional small bowel length (fSBL) was calculated using a validated formula: Plasma citrulline (µM) = 0.23 x small bowel length (cm) + 5.68 [21]. Plasma citrulline concentrations reflect the metabolic health of small bowel enterocytes. The functional small bowel length corresponds to the functional integrity or small bowel organ function.

### Human mast cell culture and degranulation assay

The LAD2 mast cells were a kind gift of Drs. Kirshenbaum and Metcalfe [22]. The HMC1.1 and HMC1.2 cells were kindly provided by Dr. Butterfield [23]. The cells were cultured according to the protocols in the Supplementary Material. Mast cell degranulation was measured by determining β-hexosaminidase (β-hex) activity as described in the Supplementary Material. Cell viability was quantified using the CyQUANT™ MTT Cell Proliferation Assay following the kit instructions (Invitrogen, Thermo Fisher Scientific).

### Statistics

Data pre-processing and statistical analyses were conducted using Microsoft Excel (Version 2308, Microsoft Corporation, USA). Patient data comprised reference or baseline and event values for chemokines, cytokines, amino acids and lipids of multiple blood samples from the same patient. For each sample two technical replicates for amino acids and lipids and three for chemokines and cytokines were measured at various time points and averaged for all further analyses. Baseline values were measured in four samples. The first two samples were collected 14 days after the MCA-vaccination event described herein. The other two baseline plasma samples were drawn approximately 7 years earlier 24 weeks apart. The suitability of these samples as baseline samples for amino acid, chemokine/cytokine and lipid analysis was recently discussed [16] and is described in more detail with specific relevancy for this study in the Supplementary Material. In general, plasma metabolome, lipidome, chemokine and cytokine data show minimal variation in the same subject over several years [15, 16]. 

Specifically, for chemokines and cytokines we compared the mean concentrations in pg/ml of two samples with or without diminazene aceturate measured in triplicate withdrawn two weeks after the MCA-vaccination event (August 2021) with the mean concentrations of two plasma samples measured in triplicate but obtained in September 2013 and March 2014 or approximately 7 years earlier with no symptoms. These samples were measured in the same Luminex experiment on the same plate. The congruency of the 28 chemokine/cytokine pairs is remarkable and confirms that within the same individual plasma samples from most chemokines and cytokines drawn 7 years apart can be used a baseline (Supplementary Figure S3 and S4). The mean (SD) absolute deviation of the mean of both means of the 28 chemokine/cytokine concentrations is only 26% (27%). For the metabolomics data the comparison between the baseline samples is more complex because 41 different parameters have been analysed but the congruency using linear regression is also excellent as described in detail in the Supplementary Table S2, Table S3 and Figure S5. The four metabolomic parameter baseline samples have been analysed in the same liquid-chromatography mass spectrometry (LC-MS) run.

Based on correlation analysis between the current and the two samples withdrawn 7 years earlier, most lipid classes cannot be readily compared (see Supplementary Table S4, Figure S6 and Figure S7). The exception are lysophosphatidylcholine (LPC) and lysophosphatidylethanolamine (LPE) lipids. Lipid resynthesis and also nutritional intake leads to increased lipid concentrations and the equilibrium concentration is not reached after two weeks, likely because of the long half-lives and slow plasma turnover of many lipids. Nevertheless, the LPC/LPE subclasses are relatively stable with a theoretical correction factor of 1.09. This subclass showed already previously substantial alterations during a severe MCA event and represents the “most interesting” lipid subclass during MCA events [16]. LPC/LPE resynthesis after degradation is almost complete after 48 h with no “overshoot” and therefore these lipids can be used for analysis [16]. Lipid parameter baseline samples have been also analysed in the same LC-MS run.

For each chemokine and cytokine a reference distribution was calculated based on the following rule. If the mean of the four means of the baseline samples measured in triplicate was above the lowest concentration of the standard curve, a reference distribution was calculated based on the four mean values. If the mean of the four means of the baseline samples was below the lowest concentrations of the standard curve, a reference distribution was constructed based on the mean and standard deviation (SD) of half of the lowest concentration of the standard curve for each substance. This is a conservative estimate because among all possible distributions with values between 0 and the lowest concentration of the standard curve, the distribution with half of the values at 0 and half at the limit has the largest variability and corresponding mean and SD of half of the lowest concentration of the standard curve. For example, if the lowest concentration of the standard curve is 4 pg/ml, then the assumption is that half of the measurements have a value of 0 and the other half 4 pg/ml, with mean and SD of 2 pg/ml. This was necessary for 15 out of the 28 chemokines and cytokines. The limit of detection (LOD) and the lowest concentration of the standard curves of the 28 chemokines/cytokines were almost identical with mean/median absolute percentage differences of 8.2 and 2.0%.

For metabolomic parameters a reference distribution was calculated based on the four baseline samples. None of the samples was below the LOD. The geometric mean and median of the ratio of the mean of the four baselines samples and the LOD is 357 and 458 respectively.

Event values were defined as measurements of blood samples drawn within 45 h of hospital admission after vaccination. Event values for amino acids, chemokines, cytokines and lipids were compared to the reference distribution using a two-sided one-sample t-test. The resulting pvalues were adjusted for multiple comparisons using the conservative Bonferroni correction accounting for the number of amino acids (*n* = 41), cytokines (*n* = 28), and lipid classes (*n* = 16). Only lipids from two lipid classes (LPC and LPE) were individually compared to their reference distribution and *p*-values were Bonferroni adjusted to the number of lipids in each class (*n* = 5). The other lipid classes were not analysed because correlation analyses indicated that the baseline values two weeks after the event were not really baseline values and therefore not suitable to use as described above. Bonferroni corrected *p*-values below 0.05 were considered statistically significant.

## Results

### Clinical symptoms and inflammation parameters after SARS-CoV-2 first mRNA-1273 booster vaccination

The main clinical time course from vaccination to hospital discharge (Fig. 1A), all drugs administered (Fig. 1B), temperature and heart rate (Fig. 1C) and five laboratory parameters (Fig. 1D) of the MCA-vaccination event are presented in Fig. 1. At admission the patient presented with fever peaking at 39.9 °C, a generalized rash, multiple vomiting episodes at home, nausea and tachycardia with a peak of 133 beats per minute fulfilling two of the four systemic inflammatory response syndrome (SIRS) criteria. During the first 18 h after admission leukocytes were elevated to approximately 11 G/L close to fulfilling a third positive SIRS criteria of 12 G/L. Baseline values in this patient are 4–6 G/L. Platelet counts dropped 26% from 186 G/L at admission to 137 G/L after 14 h.

Despite the rapid improvement of clinical symptoms after administration of metamizole and the anti-IL-6 receptor antibody tocilizumab, laboratory parameters demonstrated a delayed reaction. Three hours after metamizole and two hours after tocilizumab administration DAO, IL-6, tryptase (TRYP) and CRP were higher compared to pre-tocilizumab values. Only histamine (HA) exhibited a decrease two hours after the administration of tocilizumab. Histamine was not only measured semiquantitatively with the other amino acids as part of the metabolomics panel at CeMM (Fig. 1D) but also with the state-of-the-art Immunotech (now part of Beckman Coulter) histamine ELISA IM2015. The correlation coefficient of the values between the two assays is 94.5% with a *p*-value of 0.00041. This reduction in histamine coincided with the moderate plasma elevation of the histamine-degrading enzyme DAO.

Because DAO activity was compromised during previous severe MCA events in two mastocytosis patients indicating inactivation of DAO [24], the DAO enzymatic activity was measured in all samples in duplicate from admission to discharge. The correlation coefficient between mean DAO antigen concentrations and mean DAO activity was 99% with a *p*-value < 0.0001 indicating no compromised DAO activity during the MCA-vaccination event (data not shown). Tryptase levels peaked together with DAO and IL-6 clearly demonstrating mast cell activation. The administration of both histamine receptor 1 antagonists, 50 and 60 mg diphenhydramine within 3 h and 2 × 10 mg cetirizine, did not prevent but might have mitigated symptom development.

### Chemokinome/cytokinome, metabolome and lipidome derangements during the MCA-vaccination event

We measured 28 cytokines/chemokines, 41 metabolites, 16 lipid classes comprising 380 lipids and other parameters during the first 48 h of the MCA-vaccination event and compared them to four baseline samples from the same patient with no symptoms. Markers for hemodilution like red blood cell count, hemoglobin, tyrosine, human serum albumin and total protein concentrations showed a maximum decrease of 18% compared to baseline within the first 14 h. (Supplementary Figure S1) Baseline-normalized human serum albumin concentrations are included in the figures to visualize hemodilution. Measured biochemical parameters were not corrected for hemodilution.

### Th_2_-biased cytokines are highly elevated with minimal increases in Th_1_-biased cytokines

**Fig. 2:**
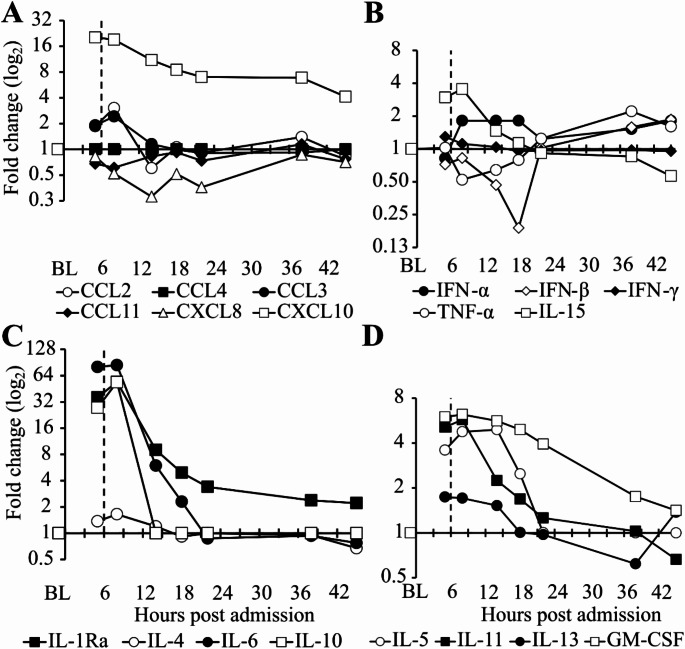
Time course of chemokine and Th_1_-/Th_2_-biased cytokine changes during mast cell activation after SARS-CoV-2 mRNA-1273 booster vaccination. The concentrations of 28 chemokines and cytokines were measured at seven time points and normalized to baseline (BL). The x-axis shows hours after hospital admission. The y-axis represents fold-changes compared to BL on a log_2_ scale. Dashed lines indicate tocilizumab administration 6 h after hospital admission. Baseline samples were obtained 2 weeks after hospital discharge. Each point represents the mean of two individual samples, citrate plasma without and with 20 µM diminazene aceturate, measured in three technical replicates. In Panel A four time points are statistically significant with *p*-values < 0.05 for CCL2 and all eight for CXCL10 with *p*-values < 0.01. In Panel B no Th_1_-biased chemokine showed statistically significant deviations. In Panel C *p*-values for IL-6 and IL-10 are below 0.001 for the first two time points. For IL-1Ra *p*-values are < 0.01 from 5 to 22 h post admission. In Panel D only GM-CSF demonstrated *p*-values below 0.05 for the first four time points. Listed *p*-values were corrected for multiple comparisons (*n* = 28)

The time courses of selected plasma chemokine and cytokine concentrations, the fold-increase and -decrease compared to the two plasma baseline samples withdrawn two weeks after the attack and the relative changes after normalization to the two baseline samples are shown in Table 1; Fig. 2.

**Table 1:** Mean baseline and event concentrations with foldchanges over baseline during the first 45 h after admission of selected chemokines and cytokines

	Concentrations in pg/ml								Foldchanges compared to BL						
Hours post admission	BL	5	8	14	18	22	38	45	5	8	14	18	22	38	45
CCL2 ^**†**^	108	**206**	**336**	**67**	117	101	**154**	93	**1.9**	**3.1**	**0.6**	1.1	0.9	**1.4**	0.9
CXCL10	20	**379**	**358**	**207**	**159**	**131**	**128**	**77**	**18.8**	**17.7**	**10.2**	**7.9**	**6.5**	**6.4**	**3.8**
IL-1Ra	425	**15,388**	**23,096**	**3849**	**2113**	**1452**	**1017**	**946**	**36.2**	**54.3**	**9.1**	**5.0**	**3.4**	**2.4**	**2.2**
IL-5	2.4	**8.8**	**12**	**12**	6.1	2.5	2.4	2.4	**3.6**	**4.8**	**4.9**	2.5	1.0	1.0	1.0
IL-6	1.4	**109**	**115**	**8.1**	3.1	1.2	1.3	1.1	**80.5**	**84.5**	**6.0**	2.3	0.9	0.9	0.8
IL-10	1.4	**39**	**76**	1.4	1.4	1.4	1.4	1.4	**27.5**	**53.9**	1.0	1.0	1.0	1.0	1.0
IL-11	140	**764**	**856**	335	252	160	153	82	**5.5**	**6.1**	2.4	1.8	1.1	1.1	0.6
GM-CSF	18	**109**	**113**	**102**	**90**	72	32	26	**6.1**	**6.4**	**5.8**	**5.0**	4.0	1.8	1.4

^†^ Only chemokines and cytokines with a least 1 significant *p*-value after Bonferroni adjustment with *n* = 28 are shown; The chemokines and cytokines with significant *p*-values over the course of the event are shown in bold and underlined; For fold-change calculations baseline (BL) plasma samples withdrawn two weeks after the event were used; Chemokine and cytokine concentrations in pg/ml are shown as the mean of the means of two blood samples measured in triplicates withdrawn with and without diminazene, a potent diamine oxidase inhibitor.

CCL2, CXCL10, IL-1Ra, IL-5, IL-6, IL-10, IL-11 and GM-CSF showed pvalues < 0.05 at least for the first two time points 5 and 8 h after hospitalization (Table 1). All pvalues have been corrected using the conservative Bonferroni method for simultaneous measurements of 28 chemokines/cytokines. Supplementary Table S1A shows the *p*-values of all time points of chemokines/cytokines with at least 1 significant *p*-value after Bonferroni correction. Twenty and 15 chemokines/cytokines did not significantly change during the first 45 h after hospitalization with or without Bonferroni correction respectively. The chemokines CCL4, CCL20 and the cytokines IL-4, IL-15 and IFNγ showed *p*-values < 0.05 after 5 h and CCL4, CCL20, IL-4 and IL-15 also after 8 h of hospital admission without Bonferroni adjustment. Except for IL-15 with a 3.5-fold peak increase over background these parameters changed less than 2-fold, which is unlikely clinically relevant.

The proinflammatory chemokine CXCL10 showed a 19-fold peak increase 5 h after hospital admission. (Fig. 2A; Table 1) The Th_1_-biased cytokines IFN-α, IFN-γ and TNF-α changed statistically nonsignificantly < 4fold during the event. (Fig. 2B) The Th_2_-biased cytokines IL-1Ra, IL-5, IL-6, IL-10 and IL-11 showed 54-, 5-, 85-, 54- and 6.1fold peak increases respectively. (Fig. 2C; Table 1) Interleukin-6 was measured in the central laboratory of the MUV with accredited protocols and also using the Luminex assay in our laboratory. Although the absolute values differed with linear regression slopes of 0.33 below 30 ng/ml IL-6 to 0.49 including all IL-6 concentrations, the correlation coefficient of all samples was almost 100%. (Supplementary Figure S2) These data indicate valid Luminex measurements.

All chemokines and cytokines except CXCL10 returned to baseline or were slightly elevated/decreased (≤ 4-fold) 22 h after hospital admission congruent with short half-lives of chemokines and cytokines and decreasing clinical symptoms (Table 1).

### Small bowel organ dysfunction and decreased amino acid levels during the MCA-vaccination event after SARS-CoV-2 mRNA-1273 booster vaccination

**Fig. 3:**
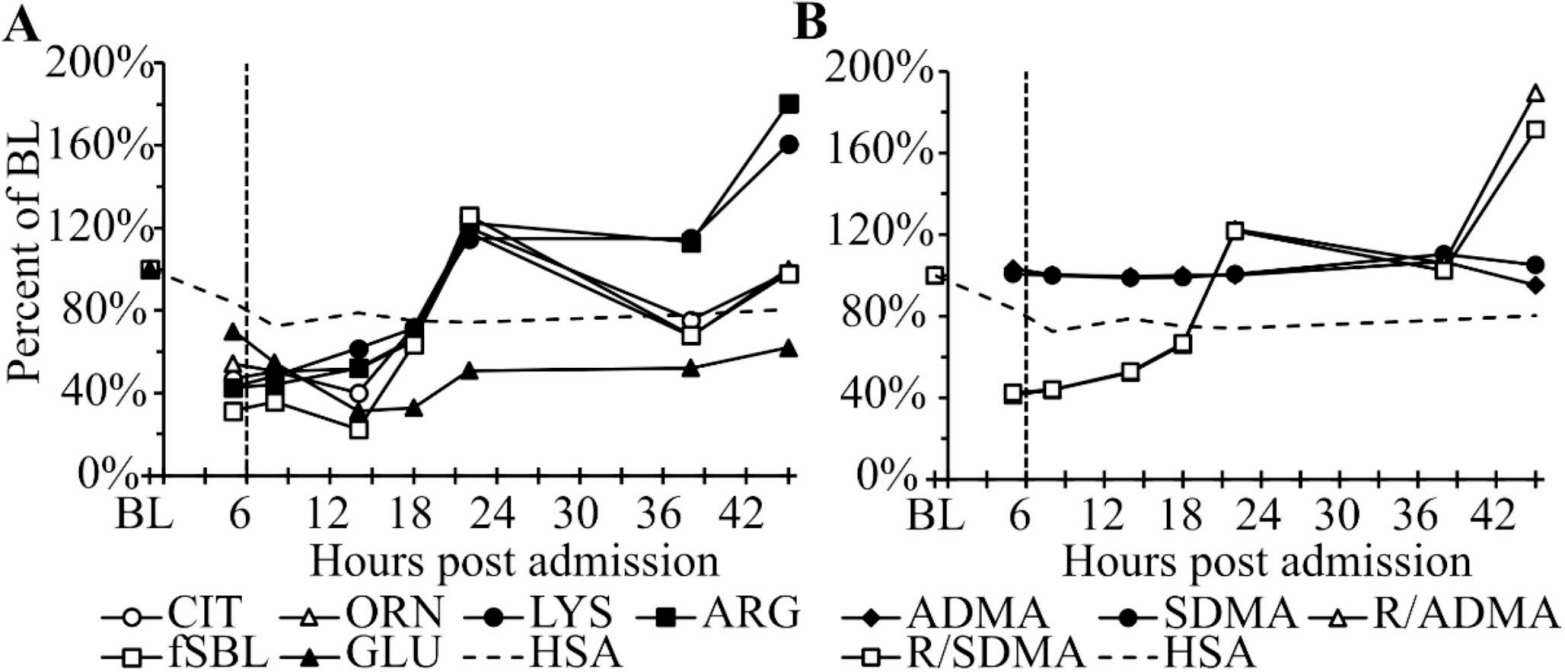
Time course of percent changes of selected metabolites during mast cell activation after SARS-CoV-2 mRNA booster vaccination. Time course of baseline (BL) normalized **A** citrulline (CIT), ornithine (ORN), lysine (LYS), arginine (ARG) and glutamic acid (GLU) concentrations and the functional small bowel length (fSBL); **B** asymmetric dimethylarginine (ADMA), symmetric dimethylarginine (SDMA), the ratio of ADMA normalized to ARG (R/ADMA) and the ratio of SDMA normalized to ARG (R/SDMA). The x-axis shows hours after hospital admission and the yaxis percent of BL values. Dashed vertical lines indicate tocilizumab administration 6 h after hospital admission. Baseline samples normalized to 100% were obtained 2 weeks after hospital discharge. Each symbol represents the mean of two citrate plasma samples without and with 20 µM diminazene aceturate. Baseline-normalized human serum albumin (HSA) concentrations are included to indicate plasma dilution. For *p*-values see text. Glutamic acid was statistically non-significantly reduced

After conservative Bonferroni *p*-value adjustment arginine concentrations statistically significantly decreased in all seven time points with pvalues between 0.04 and < 0.001. (Fig. 3A and Supplementary Table S1B) Lysine showed statistically significantly reduced concentrations 5, 8 and 14 h after admission (*p* < 0.04). Ornithine concentrations, a precursor of citrulline, was statistically significantly reduced after 8 and 14 h. Citrulline showed reduced concentrations but after Bonferroni adjustment *p*-values were above 0.05. Using only the seven time points for Bonferroni adjustment the citrulline concentrations 5, 8 and 14 h after hospitalization showed statistically significant reductions with *p*-values of 0.017, 0.02 and 0.012 respectively. Without Bonferroni adjustment the first 4 and the 6th time point showed statistically significantly reduced citrulline concentrations.

During the first 14 h the citrulline concentration-based functional small bowel length (fSBL) showed a peak 4.3-fold (77%) reduction compared to baseline (Fig. 3A). This proxy is used to describe the functional integrity of small bowel enterocytes, because these are the only cells in the human body capable of citrulline synthesis. Enterocytes in the colon produce 10-fold less citrulline and their number is smaller compared to small bowel enterocytes. The strongly reduced fSBL indicates significant small bowel dysfunction but showed a rapid recovery 22 h after admission consistent with clinical symptom improvement. Glutamine showed a strong statistically non-significant trend for reduced concentrations. (Fig. 3A)

Asymmetric (ADMA, dimethylarginine-1) and symmetric (SDMA, dimethylarginine-2) dimethylarginine, breakdown products of arginine, showed stable concentrations using baseline normalization. (Fig. 3B) Dilution effects can be avoided using the ratio of arginine to ADMA and arginine to SDMA. The decreased and consequently increased ADMA/SDMA ratios during the course of the event are caused by strongly reduced arginine concentrations. In total only 14 of the 41 measured metabolomic parameters showed in at least one time point significant increases or decreases. The *p*-values from these parameters are summarized in Supplementary Table S1B. Additional quantified metabolites are presented in the Supplementary Figure S8.

### Lysophosphatidylcholines (LPCs) are more than five-fold decreased during the first 24 h after hospital admission

**Fig. 4:**
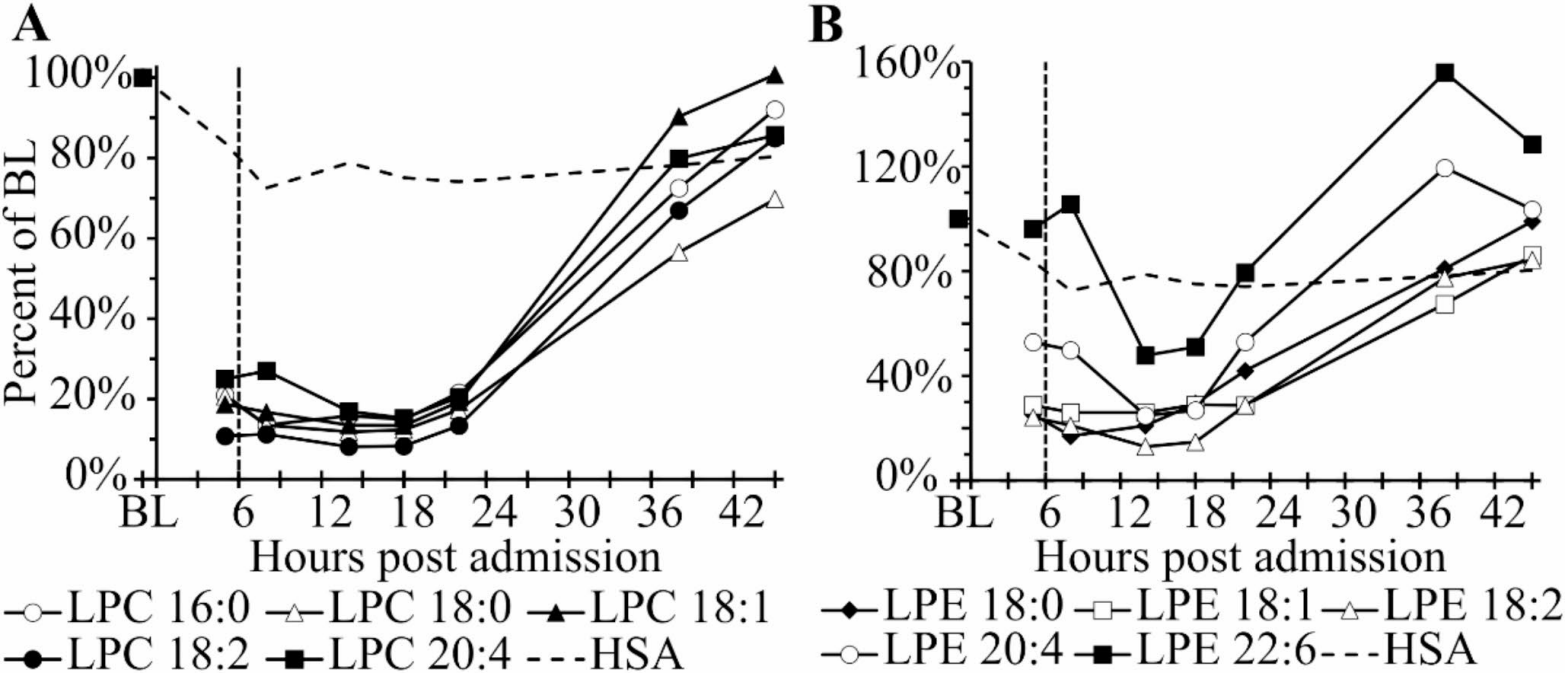
Time course of percent changes of LPC and LPE lipid variants after SARS-CoV-2 mRNA booster vaccination. Time course of baseline (BL) normalized **A** lysophosphatidylcholine (LPC) and **B** lysophosphatidylethanolamine (LPE) concentrations. The x-axis shows hours after hospital admission. Dashed vertical lines indicate tocilizumab administration 6 h after hospital admission. Baseline samples were obtained 2 weeks after hospital discharge. Each symbol represents the mean of two citrate plasma samples without and with 20 µM diminazene aceturate. BL-normalized human serum albumin (HSA) concentrations are included to represent hemodilution. The LPC baseline concentrations of the 5 lipids was 143 µM versus 4.8 µM for the 5 LPE lipids. For LPCs 92% or 23 of the 25 *p*-values during the first 22 h post admission are statistically significant after Bonferroni adjustment. 60% of the pvalues are < 0.001 (see Supplementary Table S1C)

Of the 16 measured lysophosphatidylcholine (LPC) subvariants five could be quantified without values below the LOD. They comprise 143 µM or 96% of all LPCs at baseline. We also attempted to quantify 7 lysophosphatidylethanolamine (LPE) variants and included 5 subvariants comprising 4.8 µM or 83% of baseline LPE or 3% of LPC concentrations. During the MCAvaccination event LPC and LPE variants were on average 5.6–8.1 and 2.9–4.5 fold decreased respectively between 5 and 14 h after admission (Fig. 4). The strongest maximal reduction was 12.3-fold for LPC 18:2 and 7.7-fold for LPE 18:2, both after 14 h respectively. The total detectable LPC concentration at baseline of 143 µM was reduced to only 19 µM LPCs after 18 h post hospital admission corresponding to a consumption of 125 µM or 87%. This also means that 125 µM lysophosphatidic assay (LPA) were generated likely via autotaxin activation, the enzyme responsible for LPC and LPE degradation. Recovery to baseline values was already almost complete within the following 18–36 h after hospital admission. Of the 25 values (5 LPC subvariants at 5 time points 5, 8, 14, 18 and 22 h post admission) 23 or 92% had *p*-values < 0.05 and 15 or 60% below < 0.001 after Bonferroni adjustment using *n* = 5 lipid subvariants. All *p*-values of the five selected LPC variants during the course of the event with 7 time points using *n* = 5 for Bonferroni adjustment and the *p*-values of LPC as class using *n* = 16 for Bonferroni correction are summarized in Supplementary Table S1C. Other lipid measurements are summarized in Supplementary Figure S9 and S10.

### A PCR-proven SARS-CoV-2 infection induced another MCA event in this systemic mastocytosis patient

**Fig. 5:**
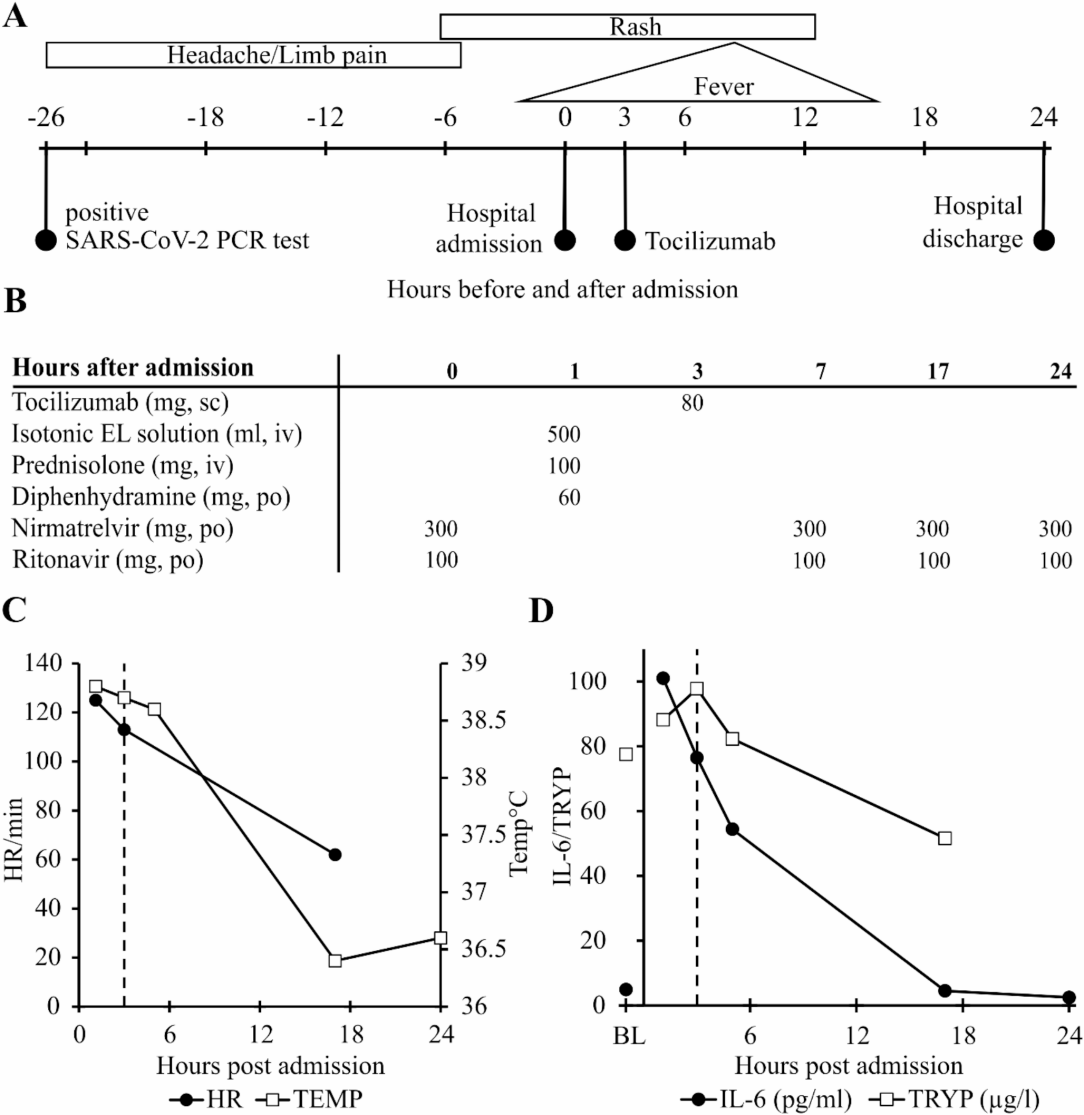
Time course of clinical symptoms, administered medications and selected clinical and laboratory parameters during a mast cell activation event likely induced by a PCR-proven SARS-CoV-2 infection. **A** Symptoms and clinical course of the MCA-infection event. **B** Medications administered after hospital admission. **C** Time course of axillary temperature (TEMP) in degrees Celsius and heart rate (HR) per minute. **D** Time course of interleukin-6 (IL-6) and tryptase (TRYP) concentrations. Baseline (BL) samples were obtained 2 weeks after hospital discharge of the MCA-vaccination event

Sixty-five weeks after the MCA-vaccination event the patient tested positive in a PCR SARS-CoV-2 assay and was admitted to the hospital 26 h later with fever, rash, multiple vomiting episodes at home and extended flush. This event also fulfilled the SIRS criteria with temperature measurements above 38 °C and a heart rate above 120 beats per minute. (Fig. 5A) After admission he received nirmatrelvir, ritonavir, prednisolone, diphenhydramine and intravenous fluids. (Fig. 5B) Because of the highly elevated serum IL-6 concentrations and the high temperature, the patient received 80 mg of tocilizumab. He did not receive metamizole. The body temperature (Fig. 5C) and IL-6 levels (Fig. 5D) normalized within the next 15 h. The patient was discharged 24 h after admission. The elevated tryptase concentrations during the course of this event clearly indicate mast cell activation. The lower tryptase concentration after 18 h has been observed in other events from the same patient (data not shown) and might be a reflection of a partial depletion of mast cell tryptase stores in this systemic mastocytosis patient after MCA. The clinical course was less severe compared to the MCA-vaccination event.

### SARS-CoV-2 mRNA vaccines induce cell death in mast cell cultures

Mast cell activation could be triggered by mRNA vaccines and therefore we tested the influence of two mRNA vaccines on the release of β-hexosaminidase as indicator of MCA. After challenging two different mast cell lines, HCM1.1 and LAD2, with the two mRNA vaccines, Comirnaty (BNT162b2, Biontech/Pfizer) and Spikevax (mRNA-1273, Moderna), a statistically not-significant trend of dosedependent degranulation was observed. The degree of degranulation varied substantially between experiments and the used cell lines. (Supplementary Figure S11) The degranulation assay (β-hexosaminidase release measurements) may be significantly influenced by cell viability. Both mRNA vaccines dose-dependently decreased cell viability rendering interpretation of β-hexosaminidase release data inconclusive. (Supplementary Figure S12)

## Discussion

Several publications describe mainly mild adverse reactions after SARS-CoV-2 vaccinations in mastocytosis patients [7–9]. The longest observation period was two hours after vaccination. Two cases of Grade 3 and Grade 4 anaphylaxis events happened among 130 adult mastocytosis patients within 30 min and might be considered “classical immediate-type” anaphylaxis [11]. In this publication we describe two MCA events in a systemic mastocytosis patient with both fulfilling SIRS criteria. The first one occurred after the first booster vaccination following priming with the same mRNA-1273 vaccine and the second one more than one year later following a PCR-positive SARS-CoV-2 infection. In the post-booster vaccination event pyrexia started as the first symptom approximately 15 h after vaccination with MCA symptoms typical for this patient of nausea, vomiting and rash presenting 24 h after vaccination. Initiation of substantial MCA was likely delayed for many hours in both events. In the more severe post-vaccination event clinical course, highly elevated IL-1Ra, IL-6 and IL-10 cytokines, LPC degradation, decreased citrulline, ornithine, arginine and lysine amino acid levels, and increased specific mast cell activation markers, histamine and tryptase, strongly support mast cell activation. We saw very similar patterns in several MCA events in this patient with no identifiable cause [15, 16]. In the PCR proven SARS-CoV-2 infection event IL-6 and tryptase concentrations were elevated strongly suggesting that the SARS-CoV-2 infection induced this MCA event.

What caused mast cell activation after the first booster vaccination and after SARS-CoV-2 virus infection? Mast cell activation after vaccination might suggest that in this patient the mRNA vaccine was able to activate his abundant mast cells possibly first locally at the injection site and consequently in other organs, based on clinical symptoms mainly skin and gastrointestinal tract. Nevertheless, similar symptoms, albeit not as severe, after SARS-CoV-2 PCRproven infection do not support this hypothesis. The mRNA vaccine hypothesis is also not supported considering that the first vaccination with the mRNA-1273 vaccine did not cause any symptoms. If it were the mRNA-1273 vaccine, we would have expected MCA symptoms after the first vaccination. It is also unlikely that this patient is unique and other mastocytosis patients might have reacted to the mRNA-1273 vaccination with a delayed MCA event.

Antibodies, likely not IgE type, might be involved in MCA, because such antibodies will be synthesized after booster vaccination but also during SARS-CoV-2 infection. Nevertheless, other mastocytosis patients should have reacted after booster vaccination or SARS-CoV-2 infection but we did not find other reports with delayed MCA in mastocytosis patients after vaccination or infection. Such events might have been assigned to the underlying disease and not to the vaccination or infection and therefore not recognized as MCA event induced after SARS-CoV-2 vaccination or during an infection. The SARS-CoV-2 virus itself is also unlikely an essential trigger considering MCA after mRNA-1273 booster vaccination, because likely thousands of mastocytosis patients have been infected with SARS-CoV-2 during the last years. A PCR-proven influenza infection caused a mild to moderate MCA event with moderately elevated Th_1_-biased cytokines in this patient [15]. The SARS-CoV-2 induced MCA event represents the second hospital-admitted virus-induced MCA incidence in this patient. Nevertheless, the T helper cell bias was not towards Th_1_ like during the influenza infection but clearly towards Th_2_. The definite trigger events in the two MCA incidents described herein are unknown.

The role of the highly elevated anti-inflammatory cytokines IL-1Ra and IL-10 is not clear. Both might reduce SIRS symptoms. Interleukin-1α and IL-1β have never been significantly elevated in these mainly Th_2_-biased MCA events and therefore the function of IL-1Ra during such events is unclear. (Data presented herein and [15]) Nevertheless, only locally but not systemically elevated IL-1 cytokines could be inhibited from binding to the IL-1 receptor biasing the response towards Th_2_. High local IL-1Ra concentrations surrounding activated mast cells will inactivate IL-1 and support immune cell activation towards Th_2_. This hypothesis is supported by showing IL-1 release at sites of antigen challenge in human cutaneous allergic reactions with concomitant mast cell activation [25]. 

It is also possible that IL-10 might be Th_1_ suppressing but Th_2_ proinflammatory via direct activation of mast cells and production of IL-5, at least in mice [26]. A positive feedback loop could be initiated, because IL-5 also primes human mast cells allowing easier activation possibly releasing more IL-10 [27]. In mice IL-10 can directly promote the activation of mast cells and plays an important pro-inflammatory role during antigeninduced mast cell activation [26].

In previous MCA events of this patient, a 5.4- to 11-fold increase of the pro-inflammatory Th_1_biased chemokine CXCL10 in plasma was observed [15]. A definite trigger in the form of a viral infection could not be established in 3 out of 4 past events with one MCA attack likely caused by a PCR-proven influenza infection. After mRNA-1273 booster vaccination CXCL10 increased 19-fold above baseline and stayed at least 4-fold elevated for 48 h. The former name of CXCL10 was IP10 or IFN-γ induced protein 10 already indicating induction by Th_1_ cytokines. This chemokine attracts mainly Th_1_ cells by binding to CXCR3. It is entirely unclear, what pathophysiological role the CXCR3/CXCL10 axis might play in Th_2_-biased MCA events.

Since IL-6 is consistently highly elevated during MCA events in this patient, the anti-IL-6 receptor antibody tocilizumab was administered in both events. Considering the pro-inflammatory role of IL-6, the strong positive correlation between IL-6 concentrations and mortality, and the adverse event profile of tocilizumab in thousands of rheumatoid arthritis patients likely treated multiple times, single administration of tocilizumab during severe MCA events is justified [28–31]. The anti-IL-6 receptor activity of tocilizumab might have been involved in the rapid reduction of the temperature in this patient, because metamizole was administered only in the MCAvaccination event. Interleukin-6 is a strong inducer of pyrexia [32]. Tocilizumab is approved for the treatment of different autoimmune diseases like rheumatoid arthritis [29], cytokine release syndrome induced by chimeric antigen receptor T-cells [33] and COVID-19 [34]. These diseases are mainly Th_1_-biased and inhibition of IL-6 is also commonly used for the treatment of Th_1_ cytokine release syndromes [13]. We are not aware of other publications using tocilizumab to treat Th_2_ cytokine release syndromes. It is tempting to speculate that tocilizumab was involved in the relatively rapid clinical improvement during both attacks. Nevertheless, it is currently undecidable, whether this patient benefited from tocilizumab administration. The decline in clinical symptoms might have just coincided with tocilizumab treatment. Generating a strong indication of causality in this patient population using a controlled, doubleblind randomized study will be challenging, to say the least. Fever reduction via tocilizumab might be of significant benefit during severe MCA events with fulfilled SIRS criteria. Sepsis patients fulfilling SIRS criteria show higher morbidity and mortality compared to non-SIRS patients [35] and this might also apply to mastocytosis patients.

Increased concentrations of IL-6 have also been measured in other systemic mastocytosis patients and uniphasic classical anaphylaxis [36, 37]. Mast cells can store IL-6 and this cytokine can be rapidly released after MCA or more slowly as recently published for a severe MCA event with possible involvement of fresh IL-6 synthesis [15, 38]. We have unpublished data from another mastocytosis patient who lost consciousness within minutes of an attack with 126 and 424 pg/ml IL-6 plasma concentrations 2 h after the first symptoms indicating storage of IL-6 in mast cells. The beneficial treatment effects of omalizumab in this patient indicates immediate-type IgEdriven MCA as cause for the attacks. Consequently, other mastocytosis patients with highly elevated IL-6 concentrations during severe MCA events might also benefit from a single administration of tocilizumab.

The chemokine/cytokine derangements are very similar in this patient with robust elevation of CXCL10, IL-1Ra, IL-5, IL-6, IL-10, IL-11 and GM-CSF during several MCA attacks [15]. There is also a consistent strong correlation between clinical symptoms, histamine concentrations and chemokine/cytokine alterations described herein and recently published [15]. Our previous speculation, also supported by data from the post-vaccination MCA event presented herein, suggests that histamine may be a key driver. Histamine was highly elevated to life-threatening concentrations 5 and 8 h after admission but was strongly decreased after 14 h. This patient suffers from skin and gastrointestinal symptoms, both organs with a high mast cell density and therefore histamine content, and degranulation of mast cells would release large amounts of histamine. It is estimated that 80% or approximately 80 mg of histamine are stored in the skin and gastrointestinal tract [16]. In mastocytosis patients with 2–10-fold elevated number of tissue mast cells, the amount of histamine stored in mast cells could be 200–1000 mg. We are not aware of data showing reduced histamine content per mast cell in mastocytosis patients compared to healthy individuals. The local histamine concentrations after MCA can reach hundreds of micromolar and at such high levels histamine is strongly pro-inflammatory, increasing vascular permeability and causing or enhancing the release of Th_2_-biased cytokines e.g. GM-CSF, IL-5, IL-6, IL-10, and IL-13 [39–42]. 

In the presented MCAvaccination event, concentrations of arginine, lysine, ornithine and citrulline were statistically significantly reduced. This pattern is reminiscent of our past report of a severe MCA event that required several days of treatment in the intensive care unit [16]. These amino acid alterations are also seen in sepsis and malaria with concomitant endothelial cell dysfunction [16]. In the MCA vaccination event the mean citrulline concentration during the first 14 h after hospital admission was 11.5 µM before returning to baseline. At citrulline concentrations below 10 µM inhospital survival of critically ill-patients decreased with an odds ratio of 8.7 indicating that the MCA-vaccination event was quite severe [43]. The reduced arginine to ADMA and arginine to SDMA ratios in the MCAvaccination event support endothelial cell distress. A two-fold ratio reduction increased mortality by approximately 30% in sepsis patients [44]. In this MCA-vaccination event both ratios decreased 5 and 8 h after hospital admission 2.3–2.4-fold before recovering 22 h after admission. Since arginine is the precursor of nitric oxide, the massive release of histamine could be involved in the plasma and endothelial cell arginine deficiency via histamine receptor activation and nitric oxide release concomitant with arginine consumption, hypoperfusion and hypoxia [16].

As citrulline is exclusively synthesized in the mitochondria of small intestinal enterocytes, it is likely that in the MCAvaccination event mast cells from the gastrointestinal tract were activated releasing large amounts of histamine and probably cytokines as described recently in more detail [16]. Enhanced vascular permeability and edema formation might induce hypoperfusion and hypoxia possibly explaining the severe reduction in the functional health of the small intestinal enterocytes. In many prior MCA attacks in this patient early symptoms are nausea and vomiting indicating mast cell degranulation in the gastrointestinal tract as an initial event. Systemically elevated histamine concentrations might induce the skin flushing and rash and might be secondary to activation of mast cells in the gastrointestinal tract or lung. Peak histamine concentrations in this post vaccination event were 15 and 18 ng/ml 5 and 8 h after admission respectively. After 14 and 18 h histamine levels were 2.6 and 0.6 ng/ml respectively with decreased chemokine and cytokine concentrations and reduced clinical symptoms. Fifteen to 18 ng/ml of circulating plasma histamine concentrations are considered life-threatening [45]. Circulating histamine concentrations represent only approximately 1% of administered histamine to healthy volunteers [46] and therefore assuming a plasma volume of 3 L roughly 4.5 mg of histamine might have been released. Considering the short plasma half-life of histamine of 2–6 min, this number is likely an underestimation [47, 48]. The massive release of histamine together with low anti-histamine tissue concentrations [49–52] might explain the inability of anti-histamines to effectively inhibit histamine during anaphylaxis or severe MCA events.

It is striking that 125 µM or 87% of the baseline LPC concentration were degraded already at the first measurement time point 5 h after hospital admission. The role of released LPA can only be speculated. Nonetheless, LPA is able to induce vascular leakage, plasma exudation and interestingly histamine release from mast cells [53, 54]. Histamine and LPA might be involved in a positive feedback loop contributing to severe hypoperfusion and hypoxia in the gastrointestinal tract [16]. It is entirely unclear, how autotaxin, the likely predominant LPC degrading enzyme, is activated to attack LPC with massive LPA release. Similar LPC degradation has been also described in sepsis, malaria and trypanosomiasis [16]. Autotaxin inhibitors are in clinical development but none are currently approved.

As previously described, glucocorticoid administration and fasting are unlikely the reason for the consistent and reproducible chemokine and cytokine, metabolomic and lipidomic derangements measured in this patient during MCA attacks ( [15, 16], Supplementary Material of this publication)

In conclusion, to the best of our knowledge we provide not only the first description of a delayed MCA event in a systemic mastocytosis patient after a SARS-CoV-2 mRNA booster vaccination but also following a PCR-proven SARS-CoV-2 infection. The chemokine and cytokine derangements show a Th_2_-biased cytokine release syndrome with highly elevated concentrations of predominantly Th_2_ cytokines concomitant with elevated histamine and tryptase levels, bona fide markers of MCA. We also describe the first time the treatment of two MCA attacks in a systemic mastocytosis patient with the anti-IL-6 receptor antibody tocilizumab. It is speculation to conclude that this patient benefited from IL-6 antagonism in addition to the likely beneficial reduction of pyrexia. Nevertheless, IL-6 concentrations can be rapidly measured and if they are highly elevated, tocilizumab administration might benefit other mastocytosis patients during severe MCA attacks.

## Electronic supplementary material

Below is the link to the electronic supplementary material.

Supplementary Material 1

## Data Availability

No datasets were generated or analysed during the current study.
