# Supplementary material for: COVID-19 mRNA-1273 vaccination induced mast cell activation with strongly elevated Th2 cytokines in a systemic mastocytosis patient
Source: Inflamm Res. 2025 Apr 29;74(1):71. doi: 10.1007/s00011-025-02032-5 (PMC12041034; doi:10.1007/s00011-025-02032-5)
Supplement: Supplementary file 1 — Supplementary Material 1 [file 11_2025_2032_MOESM1_ESM.docx]

**Supplement to**

**COVID-19 mRNA-1273 vaccination induced mast cell activation with strongly elevated Th_2_ cytokines in a systemic mastocytosis patient**

Matthias Weiss-Tessbach^1#^, Teresa Haider^2#^, Aoife Gowran^1^, Lorenz Schubert^3^, Jakob Mühlbacher^4^, Jelena Brankovic^1^, Markus Wahrmann^5^, Bernd Jilma^1^, Thomas Boehm^1^*

^1^ Department of Clinical Pharmacology, Medical University Vienna, Vienna, Austria

^2^ Division of Neuropathology and Neurochemistry, Department of Neurology, Medical University of Vienna, Vienna, Austria

^3^ Department of Medicine I, Division of Infectious Diseases and Tropical Medicine, Medical University Vienna, Austria

^4^ Department of Surgery, Medical University of Vienna, Vienna, Austria

^5^ Division of Nephrology and Dialysis, Department of Medicine III, Medical University of Vienna, Vienna, Austria

^#^Contributed equally

*Corresponding author

Thomas Boehm

Department of Clinical Pharmacology

Medical University Vienna

Waehringer Guertel 18-20, 1090 Vienna, Austria

Tel.: +43 1 40400 49580

Fax.: +43 40400 29980

Email: thomas.boehm@meduniwien.ac.at

**Supplementary Materials and Methods**

**Lipid analysis**

Twenty µl plasma was mixed with 130 µl MS-grade methanol and 20 µl of lipid internal standard solution (SPLASH® Lipidomix®, Glucosylceramide standard, Avanti Polar Lipids). After vortexing 500 µl methyl *tert*-butyl ether (MTBE) were added and the mixture was incubated in a shaker for 10 min at room temperature. Phase separation was induced by adding 130 µl MS-grade water. After 10 min of incubation at room temperature the samples were centrifuged at 1000 g for 10 min. An aliquot of 450 µl of the upper organic phase was collected and dried under nitrogen. Samples were reconstituted in 200 µl MS-grade methanol and used for lipid analysis. The LC-MS analysis was performed using a Vanquish UHPLC system (Thermo Fisher Scientific) combined with an Orbitrap Fusion™ Lumos™ Tribrid™ mass spectrometer (Thermo Fisher Scientific). Lipid separation was performed by reversed phase chromatography employing an Accucore C18, 2.6 µm, 150 x 2 mm (Thermo Fisher Scientific) analytical column at a column temperature of 35°C. The mobile phase A was an acetonitrile/water (50/50, v/v) solution containing 10 mM ammonium formate and 0.1 % formic acid. Mobile phase B consisted of acetonitrile/isopropanol/water (10/88/2, v/v/v) containing 10 mM ammonium formate and 0.1% formic acid. The flow rate was set to 400 µl per minute. A gradient of mobile phase B was applied to ensure optimal separation of the analyzed lipid species. The mass spectrometer was operated in ESI-positive and -negative mode, capillary voltage 3500 V (positive) and 3000 V (negative), vaporizer temperature 320°C, ion transfer tube temperature 285°C, sheath gas 60 arbitrary units, auxiliary gas 20 arbitrary units and sweep gas 1 arbitrary unit. Orbitrap MS scan mode at 120000 mass resolution was employed for lipid detection. The scan range was set to 250-1200 m/z for both positive and negative ionization mode. The AGC target was set to 200000 and the intensity threshold to 5000. Data analysis was performed using TraceFinder software (Thermo Fisher Scientific).

**Metabolite analysis**

Plasma samples were prepared using a hydrophobic filter plate (MultiScreen Solvinert, hydrophobic PTFE, 0.45 µm, Merck Millipore) according to the manufacturer’s instructions. Briefly, 10 µl of plasma was placed on the 96-well hydrophobic filter plate and mixed with 20 µl of an isotopically labelled internal standard mixture. 300 µl of methanol was added and the plate was shaken at 450 rpm for 20 min at room temperature. Afterwards the sample extract was collected in an attached 96-well plate by centrifuging the filter plate for 5 min at 500 g. A Vanquish UHPLC system (Thermo Fisher Scientific) coupled with an Orbitrap Fusion™ Lumos™ Tribrid™ mass spectrometer (Thermo Fisher Scientific) was used for the LC-MS analysis. The chromatographic separation was carried out on an ACQUITY UPLC BEH Amide, 1.7 µm, 2.1 x 100 mm analytical column (Waters) equipped with a VanGuard: BEH C18, 2.1 x 5 mm pre-column (Waters). The column was maintained at a temperature of 40°C and the sample injection volume was 2 µl. The mobile phase consisted of phase A 0.15% formic acid (v/v) in water and phase B 0.15% formic acid (v/v) in 85% acetonitrile (v/v), both phases with 10 mM ammonium formate. The gradient elution with a flow rate of 0.4 ml per minute was performed for a total analysis time of 17 minutes. The Orbitrap Q Exactive (Thermo Fisher Scientific) mass spectrometer was operated in a positive electrospray ionization mode, spray voltage 3.5 kV, auxiliary gas heater temperature 400°C, capillary temperature 350°C, auxiliary gas flow rate 12, sheath gas flow rate 50. The metabolites of interest were analyzed using a full MS scan mode, scan range m/z 100 to 500, resolution 120000, AGC target 1 million, maximum integration time 50 millisecond. For determination of the small molecules urea, creatinine, choline, γ-aminobutyric acid, 3-aminoisobutanoic acid, L-valine, L-proline, taurine, L-α-aminobutyric acid, sarcosine, β-alanine, L-alanine, histamine, L-threonine, glycine, L-serine and putrescine, the scan range m/z was adjusted to 50-250. The Trace Finder 4.1 software (Thermo Fisher Scientific) was used for data processing. Eight-point linear calibration curves with internal standardization and 1/x weighing were constructed for the quantification of the metabolites.

**Mast cell cultures used for measuring the effect of mRNA vaccines on degranulation**

HMC1.1 mast cells (1) were cultured in Iscove's‑modified Dulbecco's medium (Gibco) supplemented with 10% FBS, 1.2 mM α-thioglycerol, 1 x GlutaMAX and 1 x penicillin/streptomycin. HMC1.1 cells were maintained in static cultures (37°C, 5% CO_2_, 90% humidity) in non-tissue-culture treated sterile flasks. Splitting (1:5 with no centrifugation) was performed every 2-3 days to maintain cell density between 0.35-1.5 x 10^6^ cells/ml and cell viability of >90%. LAD2 cells (2) were cultured in Stem Pro‑34 medium supplemented with 2.6% nutrient supplement, 100 ng/ml recombinant human stem cell factor (R&D Systems), 2 mM glutamine and 1 x penicillin/streptomycin. LAD2 cells were also maintained in static cultures in non-tissue-culture treated sterile flasks. Splitting (1:2 with no centrifugation) was performed every 2 weeks to maintain cell density between 0.5-1×10^6^ cells/ml and cell viability of >90%.

**Measurement of mast cell degranulation**

Mast cell degranulation was measured as previously described. (3–5) Briefly, HMC-1.1 or LAD2 cells were diluted in HEPES buffer containing 0.4% BSA (HEPES+BSA) and seeded in a 96-well plate (plate 1; polystyrene, flat bottom, clear, medium binding Greiner Bio-One) at a cell density of 1x10^4^ cells/well in 90 µl HEPES+BSA. The cells were equilibrated for 10 min. Vaccines were diluted to 20 x concentration in PBS and added in a 10 µl volume. Following incubation for 15, 30 or 60 min at 37°C in 5% CO_2_ with 90% humidity the plate was centrifuged for 5 min at 450 g at 4°C. The supernatants (50 μl) were transferred to a second 96-well plate (plate 2; supernatant = released β-hex) containing the substrate solution 4-nitrophenyl N-acetyl-β-D-glucosaminide (10 mM PNAG, Sigma-Aldrich) in citrate buffer and incubated in a warm-air oven at 37°C for 90 min. The remaining cells in plate 1 were lysed with 0.1% Triton X-100 in dH_2_O and 50 μl of lysates were transferred to a third 96-well plate (plate 3; lysed = intracellular β-hex) also containing 10 mM PNAG and incubated as described for plate 2. The reaction was stopped by the addition of 50 µl of 400 mM glycine. The absorbance was measured at 405 nm (Synergy H1, BioTek). ß‑hexosaminidase release was calculated with the following formula: % degranulation = [(OD sn*df) / ((OD lys*d.f.) + (OD sn*d.f.))] × 100. (OD = optical density; sn = supernatant; df = dilution factor; lys = lysed)

**Determination of cell viability**

Cell viability and proliferation was measured using the CyQUANT™ MTT Cell Proliferation Assay (Invitrogen, Thermo Fisher Scientific). HMC-1.1 or LAD2 cells were diluted in HEPES buffer containing 0.4% BSA and seeded in a 96-well plate (polystyrene, flat bottom, clear, medium binding Greiner Bio-One) at a cell density of 1x10^5^ cells/well in 90 µl HEPES+BSA. The cells were equilibrated (10 min, 37°C, 5% CO_2_, 90% humidity) before adding treatments using 10x concentrations of investigational substances in PBS. Hydrogen peroxide and non-treated cells were used as positive and negative controls respectively. Following treatments (60 min, 37°C, 5% CO_2_, 90% humidity), 12 mM MTT (3-(4,5-dimethylthiazol-2-yl)-2,5- diphenyltetrazolium bromide) solution was added and the plate was incubated for 2 hours (37°C, 5% CO_2_, 90% humidity, protected from light). Insoluble formazan produced by the action of metabolically active cells was solubilized by adding 10% SDS in 0.01 M HCl and incubated for 18 hours in a dry oven at 37°C protected from light. The contents of each well were mixed by pipetting and absorbance was measured at 570 nm using a spectrophotometer (Synergy H1, BioTek).

**Supplementary Results**

**Chemokine/cytokine, metabolome and lipidome data after SARS-CoV-2 first booster vaccination**

**Supplementary Table S1A: Significant p-values of chemokines and cytokines after Bonferroni correction with n = 28**

| **Hours post admission** | **5** | **8** | **14** | **18** | **22** | **38** | **45** | **Panel Figure 2** |
| --- | --- | --- | --- | --- | --- | --- | --- | --- |
| **CCL2** | **0.0032** | **0.0002** | **0.0330** | 5.2439 | 2.1366 | **0.0338** | 0.4328 | A |
| **CXCL10** | **0.0000** | **0.0000** | **0.0001** | **0.0002** | **0.0004** | **0.0004** | **0.0030** | A |
| **IL-1ra** | **0.0000** | **0.0000** | **0.0002** | **0.0014** | **0.0055** | **0.0242** | **0.0337** | C |
| **IL-5** | **0.0485** | **0.0187** | **0.0168** | 0.1734 | 4.9618 | 5.0422 | 5.0422 | D |
| **IL-6** | **0.0000** | **0.0000** | **0.0089** | 0.2279 | 7.9146 | 6.3191 | 11.4178 | C |
| **IL-10** | **0.0009** | **0.0001** | 24.8264 | 24.8264 | 24.8264 | 24.8264 | 24.8264 | C |
| **IL-11** | **0.0470** | **0.0320** | 0.9022 | 2.6523 | 12.1670 | 13.6550 | 20.4321 | D |
| **GM-CSF** | **0.0190** | **0.0163** | **0.0239** | **0.0404** | 0.1019 | 7.1212 | 21.2732 | D |

**Bold and underlined** means significant with p-values <0.05 after Bonferroni adjustment.

Eight chemokines and cytokines out of 28 (29%) showed at least one statistically significant p‑value after Bonferroni correction using n = 28 (Supplementary Table S1A). The remaining 20 parameters CCL4, CCL3, CCL11, CCL20, CXCL8 (IL-8), IL-1α, IL-1β, IL-2, IL-3, IL-4, IL-13, IL-15, IL-33, IFN‑α, IFN‑β, IFN‑γ, TNF‑α, G‑CSF, bFGF and PDGF-AA did not show any significant p-value at any time point after Bonferroni correction using n = 28 (data not shown).

The chemokines and cytokines CCL4, CCL20, IL-4, IL-15 and IFN‑γ showed at least 1 significant p-value (<0.05) without Bonferroni adjustment. Nevertheless, the peak‑fold difference to baseline was less than 2-fold with the exception of IL-15 with a 3.5-fold difference.

**Supplementary Table S1B: Significant and non-significant p-values of metabolome parameters after Bonferroni correction using n = 41**

| **Hours post admission** | **5** | **8** | **14** | **18** | **22** | **38** | **45** | **UP/ DOWN** |
| --- | --- | --- | --- | --- | --- | --- | --- | --- |
| **Beta-Alanine** | 0.269 | 0.227 | 0.156 | 23.017 | **0.024** | 0.334 | 0.137 | DOWN |
| **Carnitine** | **0.009** | 0.507 | **0.005** | **0.003** | **0.004** | 0.102 | 0.105 | UP |
| **Creatinine** | 0.485 | **0.023** | 3.918 | 1.118 | 0.136 | 39,517 | 0.414 | DOWN |
| **Dimethylarginine-2** | 25.934 | 27.188 | 5.115 | 6.816 | 27.697 | **0.033** | 0.291 | UP |
| **Histamine** | **0.001** | **0.001** | 0.057 | 0.819 | 13.078 | 4,367 | 1.000 | UP |
| **Isovalerylcarnitine** | **0.000** | 27.488 | **0.001** | **0.000** | **0.000** | **0.004** | **0.000** | UP |
| **L-Arginine** | **0.001** | **0.001** | **0.001** | **0.003** | **0.008** | **0.040** | **0.000** | DOWN |
| **L-Leucine** | 2.997 | 1.285 | 0.386 | 0.103 | **0.007** | 34.023 | 0.144 | UP |
| **L-Isoleucine** | **0.017** | **0.013** | 0.103 | 0.148 | **0.003** | 4.700 | **0.036** | UP |
| **L-Lysine** | **0.012** | **0.015** | **0.034** | 0.073 | 1.738 | 1.600 | **0.015** | DOWN |
| **L-Phenylalanine** | **0.022** | **0.011** | **0.006** | **0.001** | **0.000** | 0.295 | **0.046** | UP |
| **L-Proline** | **0.035** | **0.036** | **0.030** | 0.086 | 0.385 | 0.170 | 1.122 | UP |
| **L-Tyrosine** | 2.561 | 1.131 | 2.425 | 0.410 | **0.020** | 2.240 | 0.087 | UP |
| **Ornithine** | 0.056 | **0.045** | **0.048** | 0.128 | 0.551 | 0.165 | 23.214 | DOWN |
| **Ornithine*** | **0.0096** | **0.0076** | **0.0082** | **0.0219** | 0.0940 | **0.0281** | 3.9634 | DOWN |
| **Citrulline*** | **0.0168** | **0.0199** | **0.0123** | 0.0785 | 0.8102 | 0.1066 | 1.9860 | DOWN |

*For ornithine and citrulline Bonferroni corrections using n = 7 for seven time points are also shown indicating the strong trend for reduced citrulline plasma concentrations; **Bold and underlined** p-values are statistically significant or <0.05.

Fourteen out of 41 (34%) measured metabolomics parameters were statistically significantly different during at least one time point compared to baseline values after conservative Bonferroni adjustment using n = 41. (Supplementary Table S1B) The following parameters did not show statistically significant derangements: Citrulline (see also Supplementary Table S1B using Bonferroni adjustment with n = 7), 4-hydroxyproline, aminoadipic acid, butyrylcarnitine, dimethylarginine-1, kynurenine, L-acetylcarnitine, L-alanine, L-alpha-aminobutyric acid, L-asparagine, L-cystathionine, L-cystine, L-glutamic acid, L-glutamine, L-histidine, L-methionine, L-octanoylcarnitine, L-serine, L-threonine, L-tryptophan, L-valine, methylhistidine, sarcosine, serotonin, spermidine, taurine and urea.

**Supplementary Table S1C: Significant p-values of LPC subvariants and LPC as lipid class after Bonferroni correction**

| **Hours post admission** | **LPC 16:0*** | **LPC 18:0*** | **LPC 18:1*** | **LPC 18:2*** | **LPC 20:4*** | **LPC**** | **UP/ DOWN** |
| --- | --- | --- | --- | --- | --- | --- | --- |
| **5** | **0.0157** | 0.0611 | **0.0026** | **0.0002** | **0.0004** | **0.0098** | DOWN |
| **8** | **0.0123** | **0.0493** | **0.0024** | **0.0002** | **0.0005** | **0.0090** | DOWN |
| **14** | **0.0132** | **0.0471** | **0.0022** | **0.0002** | **0.0003** | **0.0081** | DOWN |
| **18** | **0.0128** | **0.0477** | **0.0022** | **0.0002** | **0.0003** | **0.0081** | DOWN |
| **22** | **0.0160** | 0.0552 | **0.0027** | **0.0002** | **0.0003** | **0.0097** | DOWN |
| **38** | 0.2025 | 0.2290 | 0.2696 | **0.0046** | **0.0316** | 0.1409 | DOWN |
| **45** | 1.2117 | 0.4349 | 1.5914 | **0.0475** | 0.1056 | 0.5357 | DOWN |

*Bonferroni using n = 5 LPC variants and **Bonferroni using n = 16 lipid classes for p-value adjustment; **Bold and underlined** p-values are statistically significant or <0.05.

**Time course of different hemodilution markers as percent of baseline during the first 14 hours of the MCA-vaccination event**

Human serum albumin (abbreviated as HSA or A, 67 kDa) is a widely used marker for hemodilution or hemoconcentration. At the first timepoint after hospital admission during the MCA-vaccination event only a mild HSA concentration maximum decrease of 8% was measured, which increased to 18% after 14 hours. The patient received a total of three liters intravenous isotonic electrolyte solution. The time course of tyrosine (TYR or T) and of total protein (TP) concentrations was similar to HSA. Red blood count (RBC or R) or similarly hemoglobin (HB) were reduced less than 10%. (Supplementary Figure S1) The difference between mean R/HB and mean TP/A/T likely reflects increased vascular permeability induced by mast cell activation and histamine release causing “selective” diffusion of HSA and TYR into the interstitial fluid compartment. Nevertheless, because the effect is rather minor, we did not correct for hemodilution in the figures and statistical analysis but added the time course of baseline-normalized HSA concentrations to figures as appropriate.


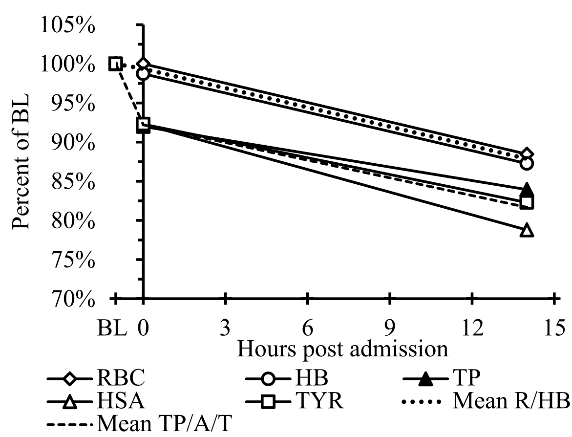


**Supplementary Figure S1:** **Time course of hemodilution markers as proxy for plasma dilution**

Time course of red blood cell count (RBC), hemoglobin (HB) and the mean of RBC and HB (R/HB) plus total plasma protein (TP), human serum albumin (HSA or A) and tyrosine (TYR or T) level and the mean of TP, HSA and TYR ((TP/A/T) are shown during the first 14 hours after hospital admission. Baseline (BL) samples for normalization were obtained 2 weeks after the hospital discharge with no clinical symptoms.

**Correlation of laboratory Luminex IL-6 measurements with IL-6 data from the Department of Laboratory Medicine at the MUV using an accredited method**

Interleukin-6 was measured at several time points using accredited methods in the clincial chemistry laboratory of the MUV but also using the Luminex platform in the research laboratory. The following figure shows the high congruency between both methods validating the laboratory Luminex measurements, at least on a relative scale. The slopes are not identical. Absolute Luminex IL-6 concentrations might be derived by multiplying with 3.0 assuming that the MUV measurements are more accurate.


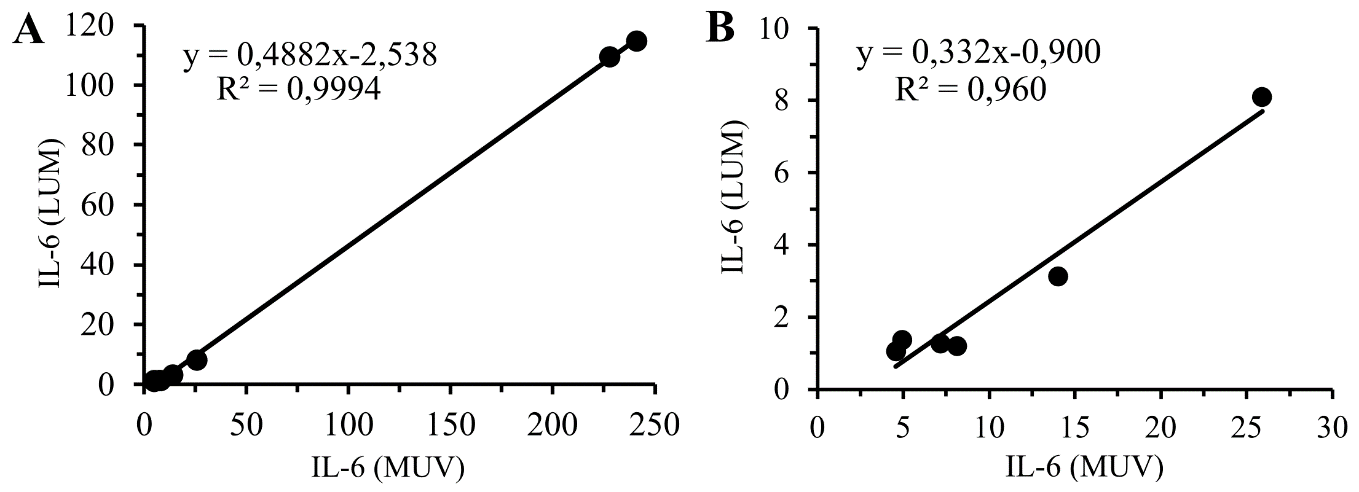


**Supplementary Figure S2:** **Comparison of Interleukin-6 (IL-6) measurements**

**(A)** Mean IL-6 Luminex (LUM) measured concentrations (pg/ml) compared to standardized and validated IL-6 concentration measurements performed by the Department of Laboratory Medicine of the MUV at the same time points. The p-value of the regression curve is 5.5x10^‑11^. **(B)** Comparison of IL-6 concentrations below 30 pg/ml between the LUM and the MUV measurements. The p-value of the regression curve is 0.0006. The correlation coefficients are > 0.96 with different slopes using the entire measured concentration range (Panel A) or the concentrations below 30 pg/ml IL-6 measured at the MUV (Panel B).

**Baseline considerations**

Chemokinome/cytokinome, metabolome and lipidome data from the same patient during several mild and severe MCA events have been recently published. (6,7) The event data in these publications have been normalized to baseline plasma values withdrawn during a study with no clinical symptoms called DAOMAST (DM_W24 and DM_W48) in 2013 and 2014. Plasma metabolome, lipidome, chemokine and cytokine data show minimal variation in the same subject over several years in agreement with published data. (6, 7 and references therein) In the MCA-vaccination event two plasma samples without and with diminazene aceturate were also derived two weeks after the MCA-vaccination attack. There are two important considerations. First, is a distance of two weeks enough to be used as baseline for metabolome, lipidome, chemokine and cytokine concentrations? Did these various parameters return to baseline after two weeks? Second, can we also use the “old” baseline data from 2013 and 2014 and include them to derive a “more reliable” and likely more conservative reference distribution for statistical analysis?

**Comparison of baseline chemokinome and cytokinome data**

Chemokine and cytokine molecules have short circulatory half-lives. During the severe MCA event described in Boehm et al. (7) most chemokines and cytokines already returned to baseline values after 72 hours. In other words, two weeks after the event the chemokine and cytokine levels should be normal, if the patient is symptom free. Below is a figure comparing the mean concentrations (+/- SD) in pg/ml of two samples with and without diminazene aceturate measured in triplicate withdrawn two weeks after the MCA-vaccination event (August 2021; labelled BL_A21_8_25AUG_mean) with the mean concentrations (+/- SD) of two plasma samples measured in triplicate but withdrawn in September 2013 (W24) and March 2014 (W48; labelled BL_DM_24_48_EDTA_mean) or approximately 7 years earlier. These samples have been measured in the same Luminex experiment on the same plate on the same day. The congruency of 28 chemokine/cytokine pairs is remarkable and confirms that within the same patient plasma samples from most chemokines and cytokines drawn 7 years apart can be used as baseline. The mean (SD) absolute deviation of the mean of both means of the 28 chemokine and cytokine concentrations is only 26% (27%). (Supplementary Figure S3 and S4) For construction of the reference distribution for statistical analysis we used all four baseline values. For normalization in the presented figures, we only used the two baseline plasma values obtained in August 2021 two weeks after the MCA-vaccination event.


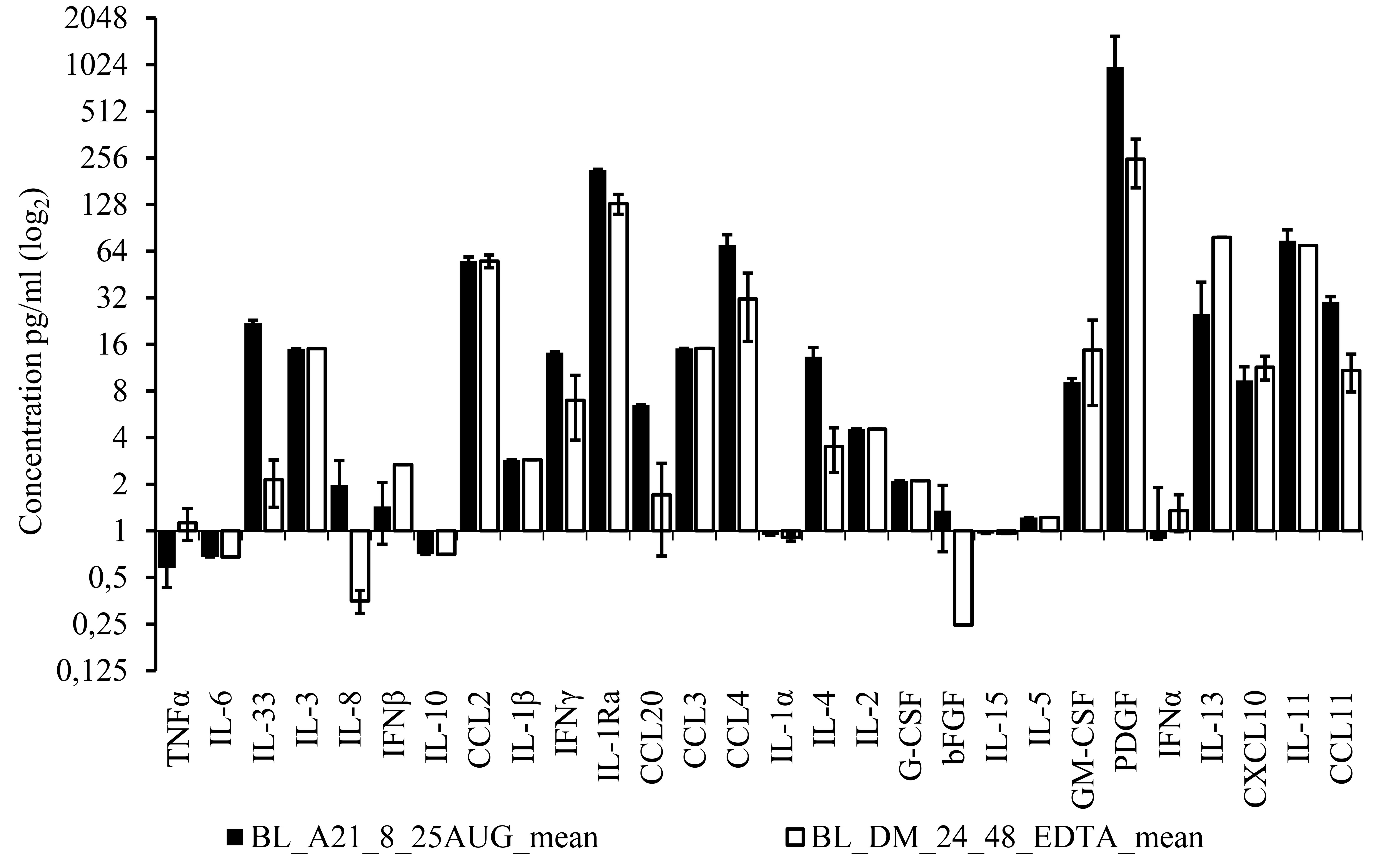


**Supplementary Figure S3: Plasma cytokine and chemokine concentrations are stable after 7 years of storage**

The mean (+/-SD) of triplicate measurements of two plasma samples drawn two weeks after the MCA-vaccination (black bars) are compared to the mean (+/-SD) of triplicate measurements of two baseline samples drawn in September 2014 and March 2014 (white bars). All four samples were measured using the Luminex platform on the same plate in 2022.


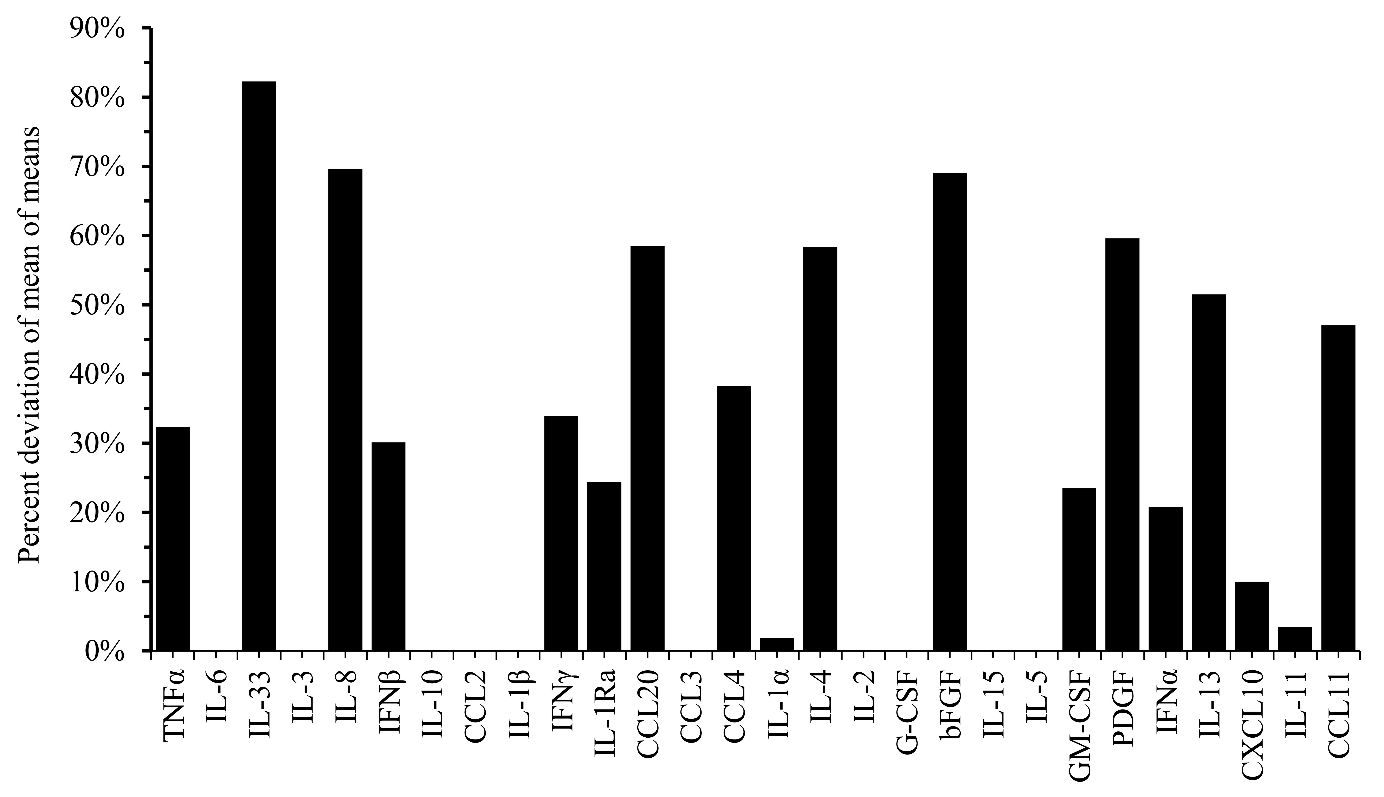


**Supplementary Figure S4: The mean absolute percent deviation of chemokine and cytokine baseline concentrations is only 26%**

The absolute percent deviation of the means of the mean of triplicate measurements of two plasma samples drawn two weeks after the MCA-vaccination and the mean of triplicate measurements of the two baseline samples drawn in September 2013 and March 2014 are shown. For example, if the IL-1Ra mean of the two MCA-vaccination baseline samples measured in triplicate is 214 pg/ml and the mean of the two IL-1Ra baseline samples from September 2013 and March 2014 is 130 pg/ml, the mean of both means is (214+130)/2 = 172 pg/ml. The absolute deviation of the two means from this mean of means is 42 pg/ml or 24%. No chemokine or cytokine baseline concentration deviates by more than 90% or more than 2-fold.

**Baseline considerations for the metabolomics data**

Metabolome data from the same patient during mild and severe MCA events have been published recently. (6) Two baselines samples drawn in September 2013 (DM_W24) and March 2014 (DM_W48) were analyzed using LC-MS at the same time with the two baseline samples with or without diminazene aceturate drawn two weeks after the MCA-vaccination event (A21). For better comparison we also grouped the metabolites into three groups: AA = Amino acids including ornithine and citrulline; AdAC = Additional amino compounds and CARN = Carnitines.

**AA (amino acid) group**: Ornithine (ORN), citrulline (CIT), alanine (ALA), arginine (ARG), asparagine (ASN), glutamic acid (GLU), glutamine (GLN), histidine (HIS), leucine (LEU), isoleucine (ILE), lysine (LYS), methionine (MET), phenylalanine (PHE), proline (PRO), threonine (THR), tryptophan (TRP), tyrosine (TYR), valine (VAL).

**AdAC (additional amino compound) group**: 4-Hydroxyproline (4-HPRO), aminoadipic acid (AAA), beta-alanine (BALA), creatinine (CREA), dimethylarginine-1 (DMARG1, equivalent to ADMA), dimethylarginine-2 (DMARG2, equivalent to SDMA), kynurenine (KYNU), l- α-amino-butyric acid (AABA), cystine (CYSTINE), methyl-histidine (Me-HIS), sarcosine (SARC), serotonin (SERO), spermidine (SPD), taurine (TAU), urea (UREA).

**CARN group:** Carnitine (CARN), isovaleryl-carnitine (ISOV-CARN), l-acetyl-carnitine (ACE-CARN), l-octanoyl carnitine (OCT-CARN).

Creatinine was not only measured at CeMM using LC-MS but also in the clincial chemistry laboratory of the MUV using an the accredited method. The correlation coefficient between the two measurements including 8 pair-wise time point data comparisons was 90% with a slope of 1.22 and a p-value of 0.0023 valdating metabolomics LC-MS measurements at CeMM (Supplementary Figure S8A).

The metabolites of the three groups and all metabolites combined were compared using simple linear regression and the main parameters are summarized in Supplementary Table S2.

**Supplementary Table S2: Results of linear regression analysis of metabolomic data comparing pairwise different baseline samples**

|  |  | **DM_24 /A21** | **DM_W48 /A21** | **DM_24 /DM_W48** |
| --- | --- | --- | --- | --- |
| **1 AA** | p-values | 4.5E-13 | 1.0E-06 | 3.8E-08 |
|  | k = | 1.0341 | 0.6933 | 0.6882 |
| n = 18 | R = | 98% | 89% | 93% |
|  | 1/k | 0.97 | 1.44 | 1.45 |
| **2 AdAC** | p-values | 3.5E-08 | 2.6E-09 | 3.7E-14 |
|  | k = | 0.7987 | 0.7339 | 0.8994 |
| n = 15 | R = | 95% | 97% | 99% |
|  | 1/k | 1.25 | 1.36 | 1.11 |
| **3 CARN** | p-values | 4.506E-10 | 1.3E-04 | 1.5E-04 |
|  | k = | 1.0743 | 0.9679 | 0.9004 |
| n = 6 | R = | 100% | 99% | 99% |
|  | 1/k | 0.93 | 1.03 | 1.11 |
| **4 ALL** | p-values | 7.209E-27 | 3.873E-17 | 4.7E-21 |
|  | k = | 1.0280 | 0.7715 | 0.7573 |
| n = 39 | R = | 98% | 93% | 95% |
|  | 1/k | 0.97 | 1.30 | 1.32 |
|  | **Mean (1-3)** | **1.05** | **1.28** | **1.23** |
|  | **SD (1-3)** | **0.18** | **0.22** | **0.20** |
|  | **CV** | **17%** | **17%** | **16%** |

Abbreviations: AA: Amino acids, AdAC: Additional amino compounds, CARN: carnitines, k: slopes of linear regression curves, R: correlation coefficient, 1/k: reciprocal slope or correction factor, SD: standard deviation, CV: coefficient of variation.

The increased slope and reduced correlation coefficient (R) values in the AA group is mainly driven by the concentrations of three amino acids GLU, GLN and MET with significant deviations in the DM_W48 sample. After removing the GLU, GLN and MET data the slope changed minimally from 1.034 to 1.019 in the DM_W24/A21 comparison but increased substantially in the DM_W48/A21 and DM_W24/DM_W48 groups from 0.694 and 0.688 to 0.876 and 0.894 respectively (data not shown). The AA group is the relevant group in the analysis in the main manuscript. The µM and percent deviations of CIT, ORN, LYS and ARG, deranged metabolites during the severe MCA event recently published (6), is minimal in the three samples (Supplementary Table S3). We also included TYR in the analysis, because it is a known stable amino acid in plasma. Phenylalanine (PHE) was also added, because it increases during stress conditions like sepsis and MCA events. All four baseline samples were used to construct the reference distribution for metabolome statistical analysis. In the figures in the main manuscript and the Supplementary Material only the mean of the two A21 plasma samples withdrawn two weeks after the MCA-vaccination event were used for baseline-normalization.

**Supplementary Table S3: Minimal deviations of ornithine, citrulline, arginine, lysine, tyrosine and phenylalanine plasma concentrations in baseline samples drawn 7 years apart**

| **Metabolite** | **A21 (µM)** | **DM_W24 (µM)** | **DM_W48 (µM)** | **Mean (µM)** |
| --- | --- | --- | --- | --- |
| ORN | 86.3 | 87.3 | 77.4 | 83.7 |
| CIT | 25.1 | 31.3 | 25.4 | 27.3 |
| ARG | 70.8 | 68.3 | 71.7 | 70.3 |
| LYS | 207.3 | 231.3 | 217.9 | 218.8 |
| TYR | 53.6 | 47.4 | 46.1 | 49.0 |
| PHE | 70.0 | 77.0 | 67.2 | 71.4 |
| **Percent of the mean** |  |  |  |  |
| ORN | 103% | 104% | 93% |  |
| CIT | 92% | 115% | 93% |  |
| ARG | 101% | 97% | 102% |  |
| LYS | 95% | 106% | 100% |  |
| TYR | 109% | 97% | 94% |  |
| PHE | 98% | 108% | 94% |  |
| **Mean percent** | **99.7%** | **104.4%** | **95.9%** |  |

ORN = ornithine, CIT = citrulline, ARG = arginine, LYS = lysine, TYR = tyrosine and PHE = phenylalanine. A21 is the mean of two plasma samples without and with diminazene aceturate withdrawn two weeks after the MCA-vaccination event in August 2021. DM_W24 and DM_W48 are plasma samples withdrawn 7 years earlier. All samples have been measured in the same metabolomics experiment.

Supplementary Figure S5 shows the linear regression curves including all metabolites (n = 39) included in the Supplementary Table S2.


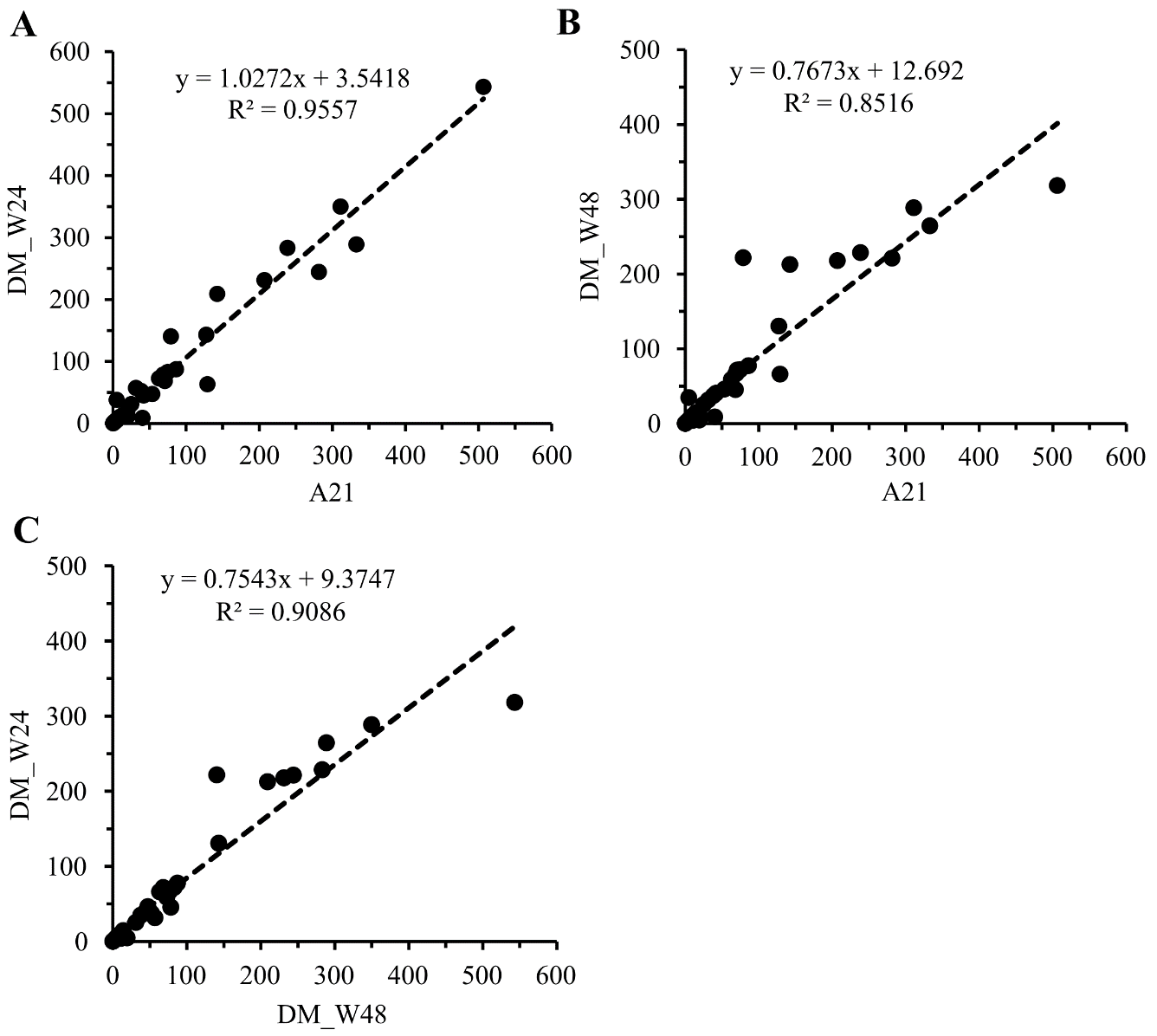


**Supplementary Figure S5: Linear regression curves of all metabolite data comparing the three baseline samples**

Metabolomics data from three different baseline plasma samples DM_W24, DM_W48 and A21 were compared using linear regression analysis. The results are summarized in Supplementary Table S2 under group ALL with n=39. The units for the x- and y-axes are µM plasma concentrations.

**Baseline considerations for the lipidomics data**

To compare the A21 lipidomic results with previous lipidomic measurements, lipid concentrations of two baseline samples 24 weeks apart (DM_W24 and DM_W48) obtained 7 years earlier were also measured in the same experiment with the new samples A21 with or without diminazene obtained 2 weeks after the MCA-vaccination event. The detected lipids from the four baseline samples are plotted using linear regression and the key data are summarized in Supplementary Table S4. The mean of the two A21 samples was used for comparison with DAOMAST baseline samples DM_W24 and DM_W48.

The correction factors (1/slope of the regression curve) are graphically presented in Supplementary Figure S6. DM_W48 compared to A21 clearly showed higher variability with a two-fold increased mean correction factor compared to DM_W24 and A21. Both the standard deviation and coefficient of variation (CV) are at least 50% lower in the DM_W24/A21 pair compared to the DM_W48/A21 pair. Nevertheless, congruency of A21 with DM_W24 is reduced compared to metabolomics data with a mean correction factor of 1.63. Nevertheless, the LPC/LPE subclasses seems relatively stable with a theoretical correction factor of 1.09 and this subclass showed already previosuly substantial alterations during a severe MCA event and seems to represent the “most interesting” lipid subclass during MCA events. (6) The LPC/LPE values showed strong reductions in previous MCA events in this patient. (6) The LPC/LPE subclasses recovered from a 4 to 8-fold decrease during the acute phase of a severe MCA event within 72 hours indicating fast resynthesis of plasma LPC/LPE lipids. (6)

Another consideration is whether the A21 baseline drawn two weeks after the MCA-vaccination event is a reliable baseline for lipid analysis. We hypothesize that the reduced concentrations of many lipids, eg DAG and TAG, is an artefact because of increased lipid synthesis as rebound after the stressful MCA-vaccination event and the long half-life of lipids of several days in plasma. Therefore, we believe that the concentrations of many lipids 2 weeks after the MCA-vaccination event do not correctly represent the true baseline values and the deviation is reflected in partially very high correction factors of 4.5 or even 6. (Supplementary Table S4; Supplementary Figure S6) As demonstration baseline‑normalized LPC, DAG and TAG concentrations have been corrected using the inverse of the slope from the regression analysis of 1.09, 2.18 and 2.25 respectively (Supplementary Table S4; Supplementary Figure S7). Based on these considerations only LPC/LPE data were statistically analysed and all baseline values were used to construct the reference distribution (A21, DM_24W and DM_48W).

**Supplementary Table S4: Results of linear regression analysis of lipid subclasses comparing different baseline sample pairs**

|  |  | **DM_W24 /A21** | **DM_W48 /A21** | **DM_W24 /DM_48** |
| --- | --- | --- | --- | --- |
| **CE, CHOL** | k = | 0.73 | 0.33 | 0.54 |
| **n = 5** | R = | 98% | 43% | 52% |
|  | 1/ k | 1.37 | 3.0 | 1.9 |
| **Cer, GlcCer** | k = | 0.75 | 0.59 | 0.77 |
| **n = 16** | R = | 98% | 43% | 52% |
|  | 1/ k | 1.34 | 1.7 | 1.3 |
| **LPC/LPE** | **k =** | **0.92** | **1.34** | **1.44** |
| **n = 18** | **R =** | **100%** | **99%** | **98%** |
|  | **1/ k** | **1.09** | **0.7** | **0.7** |
| **PC** | k = | 0.62 | 0.40 | 0.65 |
| **n = 28** | R = | 100% | 98% | 99% |
|  | 1/ k | 1.61 | 2.5 | 1.5 |
| **PC-O** | k = | 0.65 | 0.22 | 0.36 |
| **n = 19** | R = | 99% | 83% | 88% |
|  | 1/ k | 1.54 | 4.5 | 2.8 |
| **PE** | k = | 0.57 | 0.39 | 0.77 |
| **n = 35** | R = | 100% | 100% | 96% |
|  | 1/ k | 1.77 | 2.6 | 1.3 |
| **PE-O** | k = | 0.75 | 0.56 | 0.77 |
| **n = 6** | R = | 98% | 90% | 96% |
|  | 1/ k | 1.33 | 1.8 | 1.3 |
| **PI** | k = | 0.4601 | 0.168 | 0.3666 |
| **n = 4** | R = | 99.8% | 98.4% | 99.04% |
|  | 1/ k | 2.17 | 5.95 | 2.73 |
| **PS** | k = | 0.6328 | 0.3585 | 0.5996 |
| **n = 4** | R = | 98.2% | 87.2% | 93.97% |
|  | 1/ k | 1.58 | 2.79 | 1.67 |
| **SM** | k = | 0.78 | 0.74 | 0.95 |
| **n = 21** | R = | 100% | 100% | 100% |
|  | 1/ k | 1.28 | 1.3 | 1.1 |
| **TAG** | k = | 0.44 | 0.30 | 0.68 |
| **n = 48** | R = | 98% | 97% | 99% |
|  | 1/ k | 2.25 | 3.3 | 1.5 |
| **DAG** | k = | 0.46 | 0.30 | 0.68 |
| **n = 3** | R = | 100% | 93% | 94% |
|  | 1/ k | 2.18 | 3.3 | 1.5 |
|  | **mean** | 1.63 | 2.79 | 1.60 |
|  | **SD** | 0.389 | 1.415 | 0.612 |
|  | **CV** | 24% | 51% | 38% |

Abbreviations: CE: cholesteryl ester, CHOL: cholesterol, Cer: ceramide, GlcCer: Glucosylceramide, LPC: lysophosphatidylcholine, LPE: lysophosphatidylethanolamine, PC: phosphatidylcholine, PC-O: alkyl ether variants of phosphatidylcholine, PE: phosphatidylethanolamine, PE-O: alkyl ether variants of phosphatidylcholine, PI: phosphatidylinositol, PS: phosphatidylserine, SM: sphingomyelin, TAG: triacylglyceride, DAG: diacylglyceride, k: slope of different linear regression models, R: correlation coefficient, 1/k: reciprocal slope, SD: standard deviation, CV: coefficient of variation.


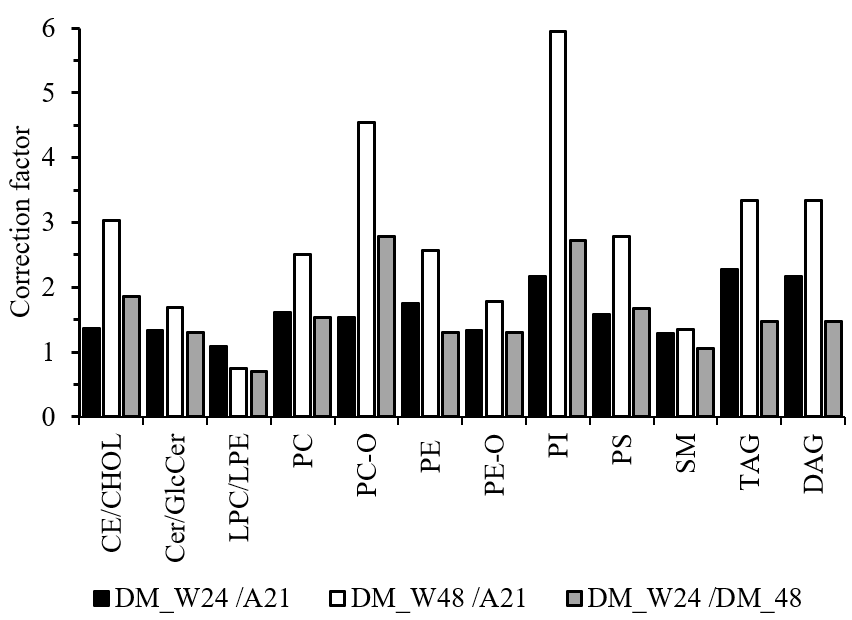


**Supplementary Figure S6: Corrector factors from Supplementary Table S4**

The influence of applying the baseline correction factor as described above is shown in the next figure. The time course of baseline‑normalized not-corrected (Panels A, C and E) and corrected LPC, TAG and DAG (Panels B, D and F) concentrations during the MCA-vaccination event are compared. (Supplementary Figure S7)


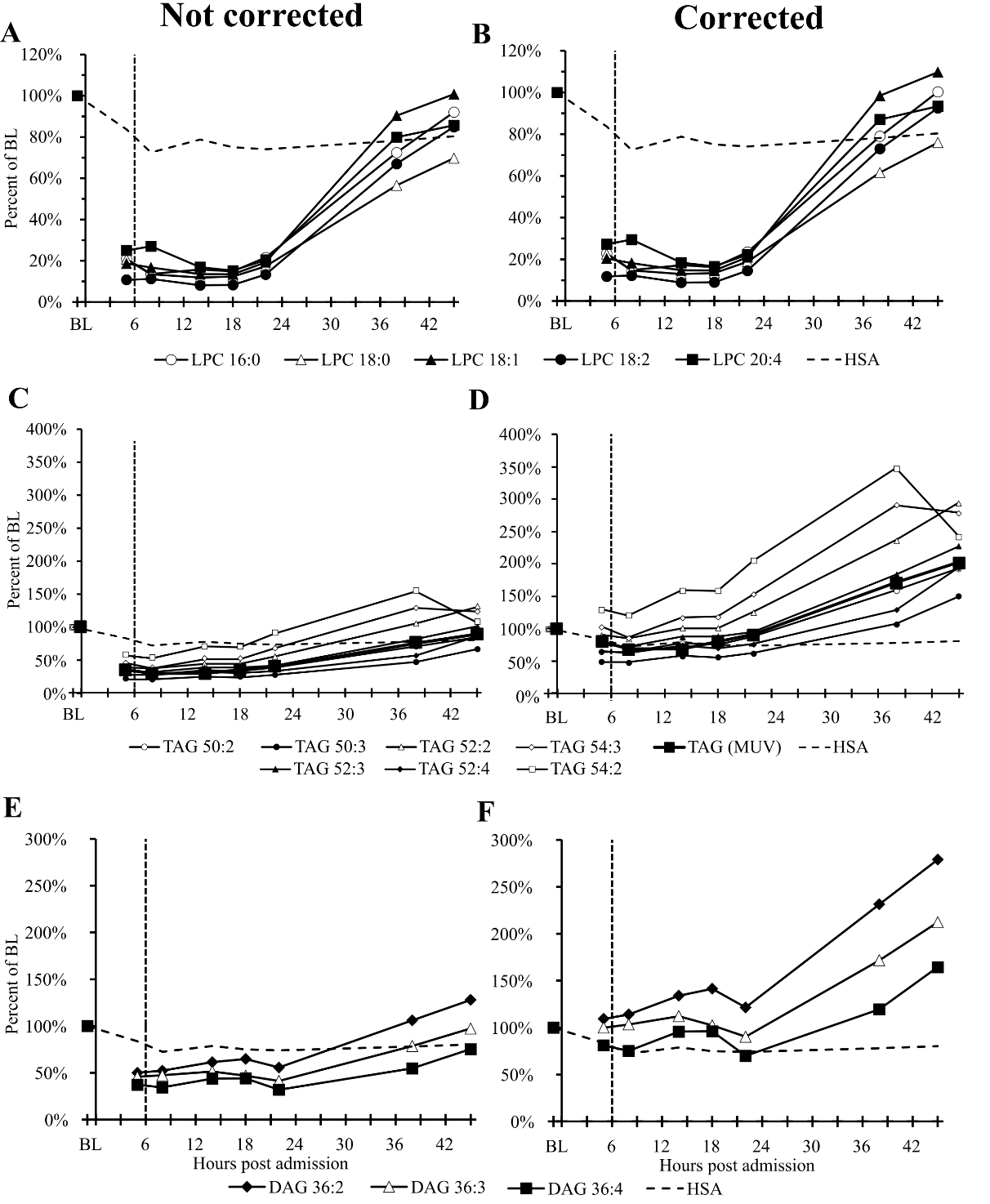


**Supplementary Figure S7: Time course of LPC, TAG and DAG baseline-normalized percent concentrations with or without applying a correction factor**

Time course of not-corrected **(A)** and corrected **(B)** lysophosphatidylcholine (LPC) variants, representing 100% of all measured LPC variants or 143 µM. Eighteen hours after admission only 19 µM LPCs or 13% were left. Time course of selected not-corrected **(C)** and corrected **(D)** triacylglyceride (TAG) variants representing 56% of all detected TAG variants. All measured TAGs correspond to a sum of 1.9 mM. The filled squares represent the change of TAGs measured by the routine clinical laboratory of the MUV. Time course of not-corrected **(E)** and corrected **(F)** diacylglyceride (DAG) variants representing 100% of 105 µM of all measured DAGs. In Panels B, D and F the correction factors of 1.09, 2.25 and 2.18 were used for LPCs, TAGs and DAGs respectively (Supplementary Table S4). Baseline (BL; 100%) samples were obtained 2 weeks after hospital discharge. Each point represents the mean lipid concentration in two citrate plasma samples with or without 20 µM diminazene aceturate. BL-normalized human serum albumin (HSA) concentrations are included.

**Additional metabolomics data**


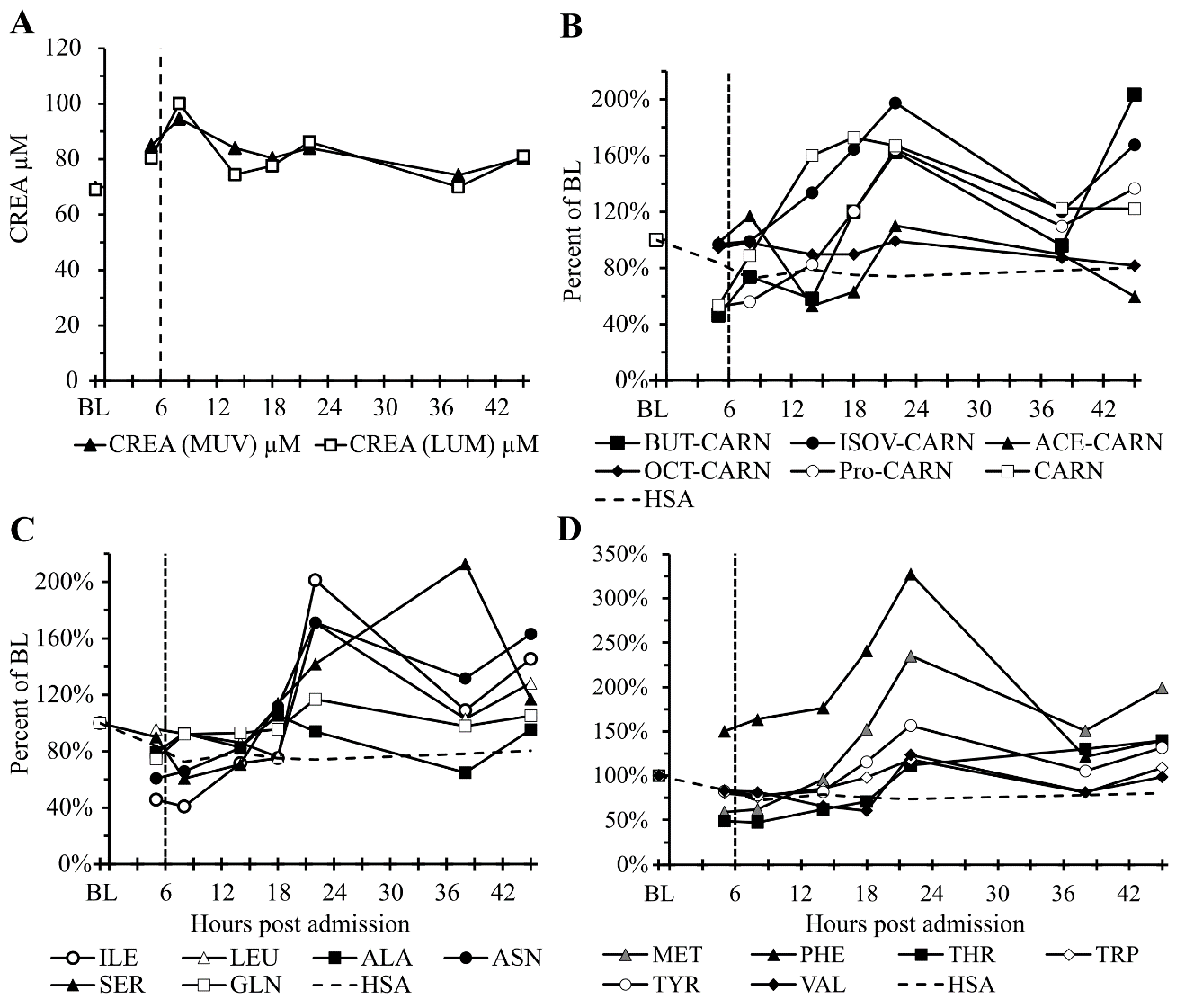


**Supplementary Figure S8: Time course of creatinine (CREA), carnitine metabolites and additional metabolomics data during the MCA-vaccination event after SARS-CoV-2** **mRNA booster vaccination**

**(A)** Time course of creatinine (CREA) concentrations (µM) using the Luminex assay (LUM) and the method from the central laboratory at the MUV. Time course of baseline (BL)-normalized concentrations in **(B)** of butyryl-carnitine (BUT-CARN), carnitine (CARN), isovaleryl-carnitine (ISOV-CARN), acetyl-carnitine (ACE-CARN), octanoyl‑carnitine (OCT-CARN) and propionyl‑carnitine (Pro-CARN); **(C)** of isoleucine (ILE), leucine (LEU), alanine (ALA), asparagine (ASN), glutamic acid (GLU) and glutamine (GLN); **(D)** of methionine (MET), phenylalanine (PHE), threonine (THR), tryptophan (TRP), tyrosine (TYR) and valine (VAL). BL-normalized human serum albumin (HSA) concentrations are included to represent hemodilution. The x-axis shows hours after hospital admission. Dashed vertical lines indicate tocilizumab administration 6 hours after hospital admission. Each symbol represents the mean of two citrate plasma samples with or without 20 µM diminazene aceturate. The two BL samples were obtained 2 weeks after hospital discharge.

**Additional lipidomics data**


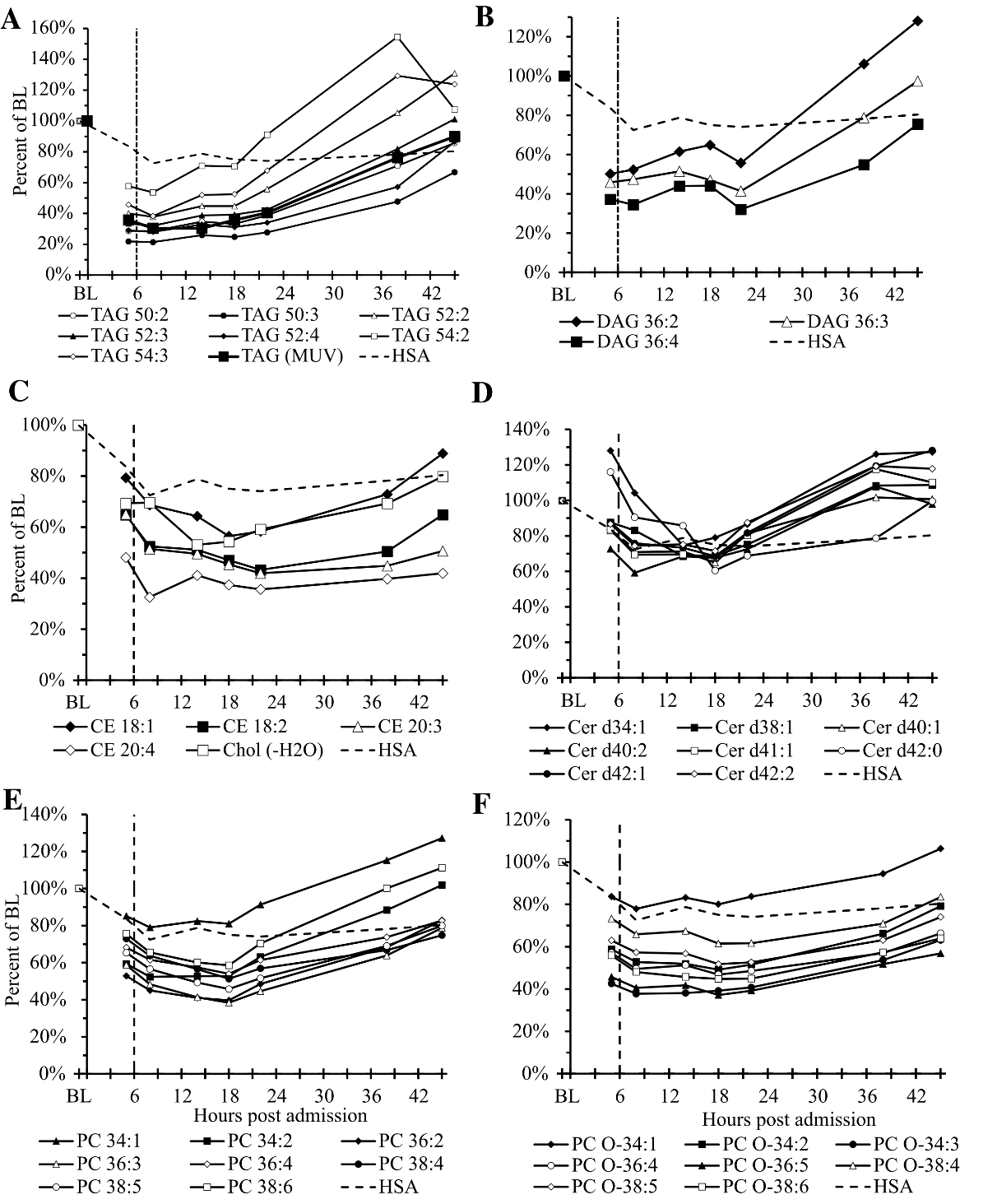


**Supplementary Figure S9: Time course of the baseline-normalized concentrations of TAG, DAG, CE, Cer, PC and their alkyl ether (PC‑O) lipid variants during the MCA-vaccination event after SARS-CoV-2 mRNA booster vaccination**

Time course of **(A)** selected triacylglycerides (TAG) representing 55.5% of all detected (above the LOD at all time points) TAGs. All measured TAGs correspond to 1.9 mM at baseline (BL). The filled squares represent TAG concentrations measured by the routine clinical laboratory of the MUV; **(B)** diacylglycerides (DAG; total 105 µM at BL); **(C)** four cholesteryl esters (CEs) and cholesterol (-H2O) representing 8.1 mM at BL; **(D)** ceramides (Cer, total 7.7 µM at BL); **(E)** phosphatidylcholines (PC) representing 89% or 1.4 mM at BL of all PCs. **(F)** Time course of detected alkyl ether variants of PCs (PC-O; 81 µM) representing 82% of all PC-Os at BL. BL-normalized human serum albumin (HSA) concentrations are included to represent hemodilution. The x-axis shows hours after hospital admission. Dashed vertical lines indicate tocilizumab administration 6 hours after hospital admission. Each symbol represents the mean of two citrate plasma samples with or without 20 µM diminazene aceturate. The two BL samples were obtained 2 weeks after hospital discharge. The BL-normalized concentrations have **not been corrected** with a correction factor as described in the text.


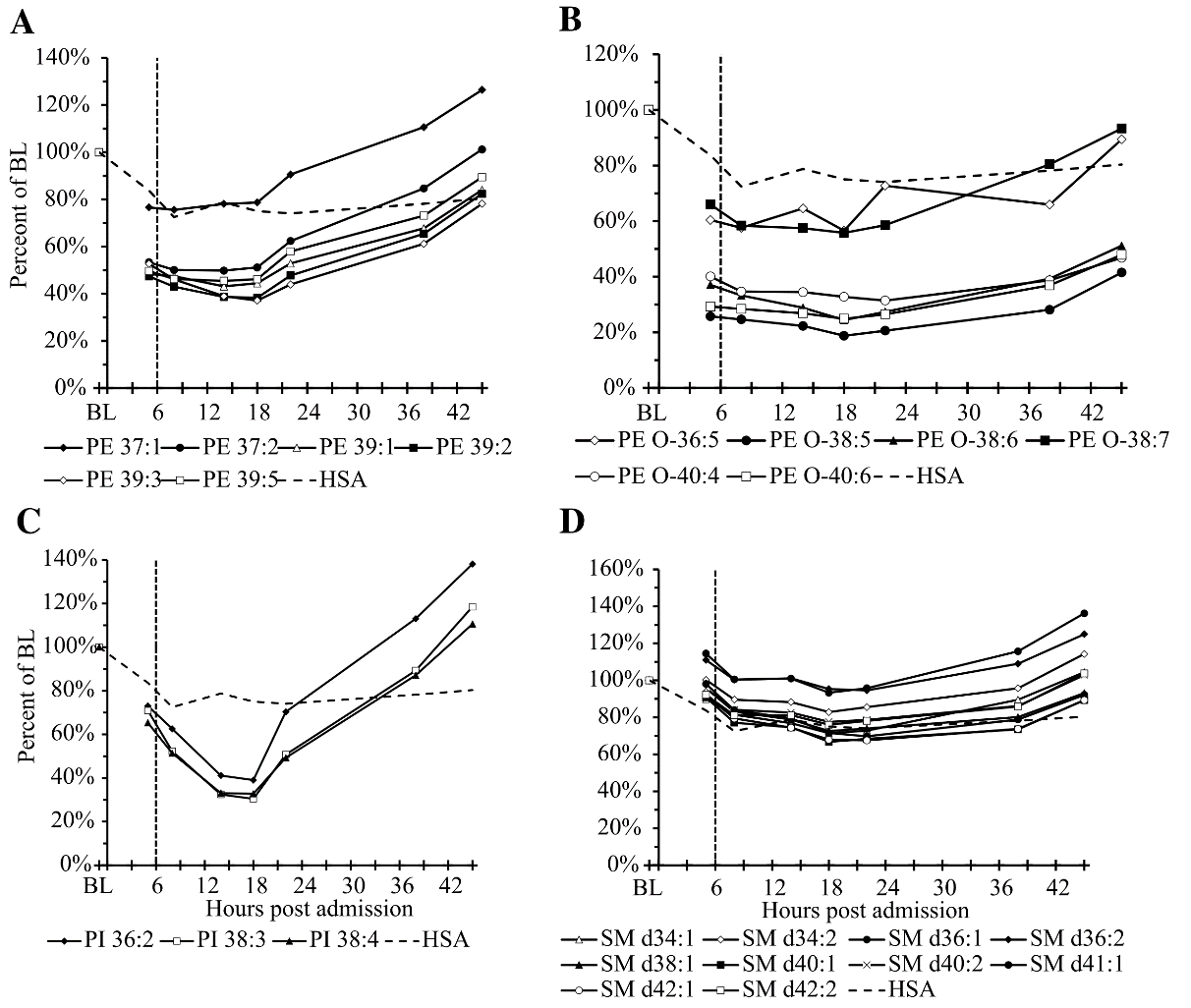


**Supplementary Figure S10: Possibly decreased levels of PI but only moderate changes of PE, PE-O, and SM lipid variants during the MCA-vaccination events after SARS-CoV-2 mRNA booster vaccination**

Time course of **(A)** selected phosphatidylethanolamines (PE) representing 93% of all PEs with a total of 2.3 mM PEs at baseline (BL); **(B)** six detected PE alkyl ether variants (PE‑O; 35 µM at BL); **(C)** three detected phosphatidylinositol variants (PI; 53 µM); **(D)** detected sphingomyelin variants (SM; 268 µM) representing 87% of all SMs at baseline. BL-normalized human serum albumin (HSA) concentrations are included to represent hemodilution. The x-axis shows hours after hospital admission. Dashed vertical lines indicate tocilizumab administration 6 hours after hospital admission. Each symbol represents the mean of two citrate plasma samples with or without 20 µM diminazene aceturate. The two BL samples were obtained 2 weeks after hospital discharge. The BL-normalized concentrations have not been corrected with a correction factor as described in the text.

**Degranulation and viability of mast cell lines after stimulation with different mRNA vaccines**

Mast cell degranulation can be initiated by various triggers like IgE/Fcε-receptor interactions, pharmaceutical agents like vancomycin or morphine, complement fragments, peptides and environmental factors like cold, heat, pressure or stress but many events cannot be assigned to a definite trigger. (8)

In vitro experiments challenging two different mast cell lines with different dilutions of mRNA vaccines (1/3, 1/6, 1/9) did not really show a significant trend towards higher degranulation using higher mRNA vaccine concentrations. (Supplementary Figure S11) Quantification of in vitro degranulation is based on the difference between the extracellular and total (intracellular + extracellular) concentrations of β-hexosaminidase. The extracellular and intracellular concentrations of β‑hexosaminidase are likely influenced by decreased cell-viability caused by the vaccine.

We performed the MTT assay to assess cell viability and indirectly cell death. After challenging cells with different mRNA vaccines, we measured a vaccine dose-dependent reduced cellular viability comparable to the effects of the positive control hydrogen peroxide (H_2_O_2_). Considering the cytotoxic effect of mRNA vaccines in vitro, the results of the β‑hexosaminidase assay cannot really be interpreted.


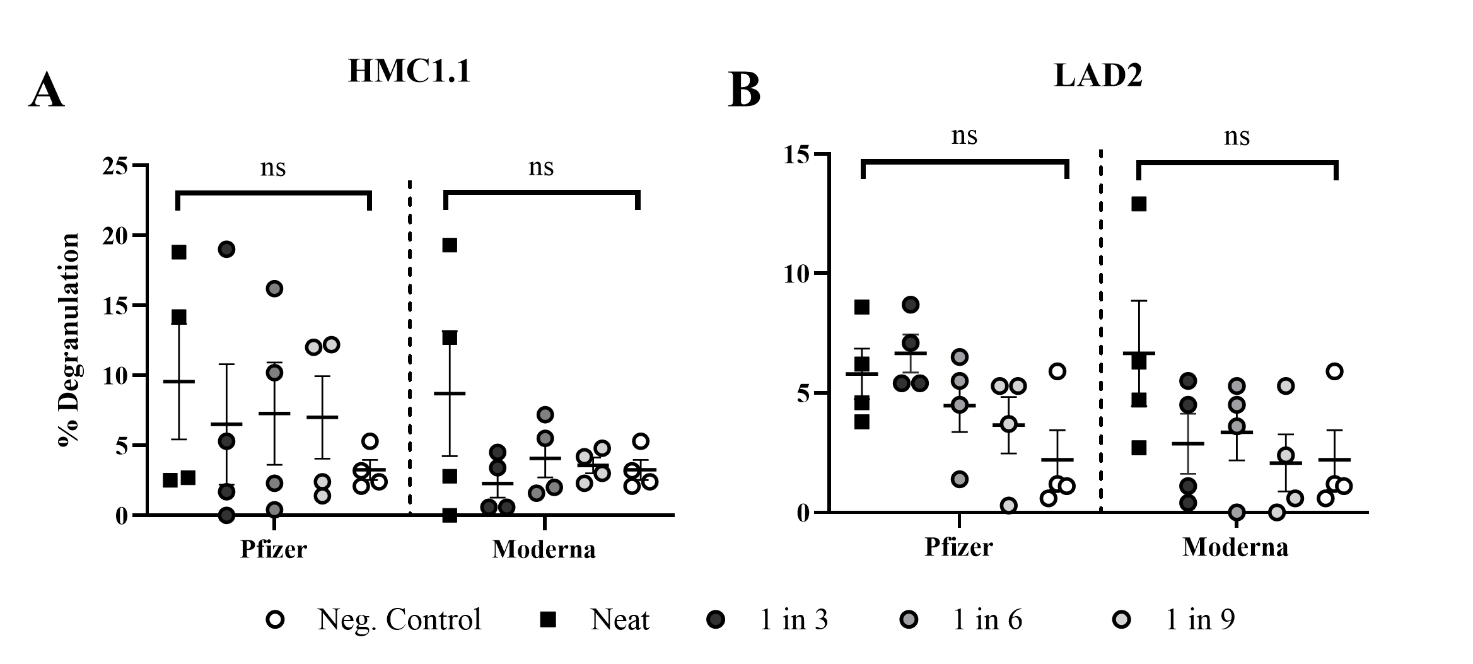


**Supplementary Figure S11: In vitro degranulation of mast cells after challenge with mRNA vaccines**

Percent degranulation of HMC1.1 **(A)** and LAD2 **(B)** cells after stimulation with Comirnaty (BNT162b2, Biontech/Pfizer) and Spikevax (mRNA-1273, Moderna). The mast cell lines HMC1.1 and LAD2 were treated with the mRNA-vaccines or buffer for one hour. Degranulation was determined using the β-hexosaminidase assay. Each point represents one individual experiment with technical duplicates and normalized to the respective blank equivalent to cells without treatment. Each substance was tested on four different days. Results are presented as individual measurements with their respective mean (± SEM). Dilution rows were tested for a linear trend using an ordinary one-way ANOVA. Neat = no dilution.


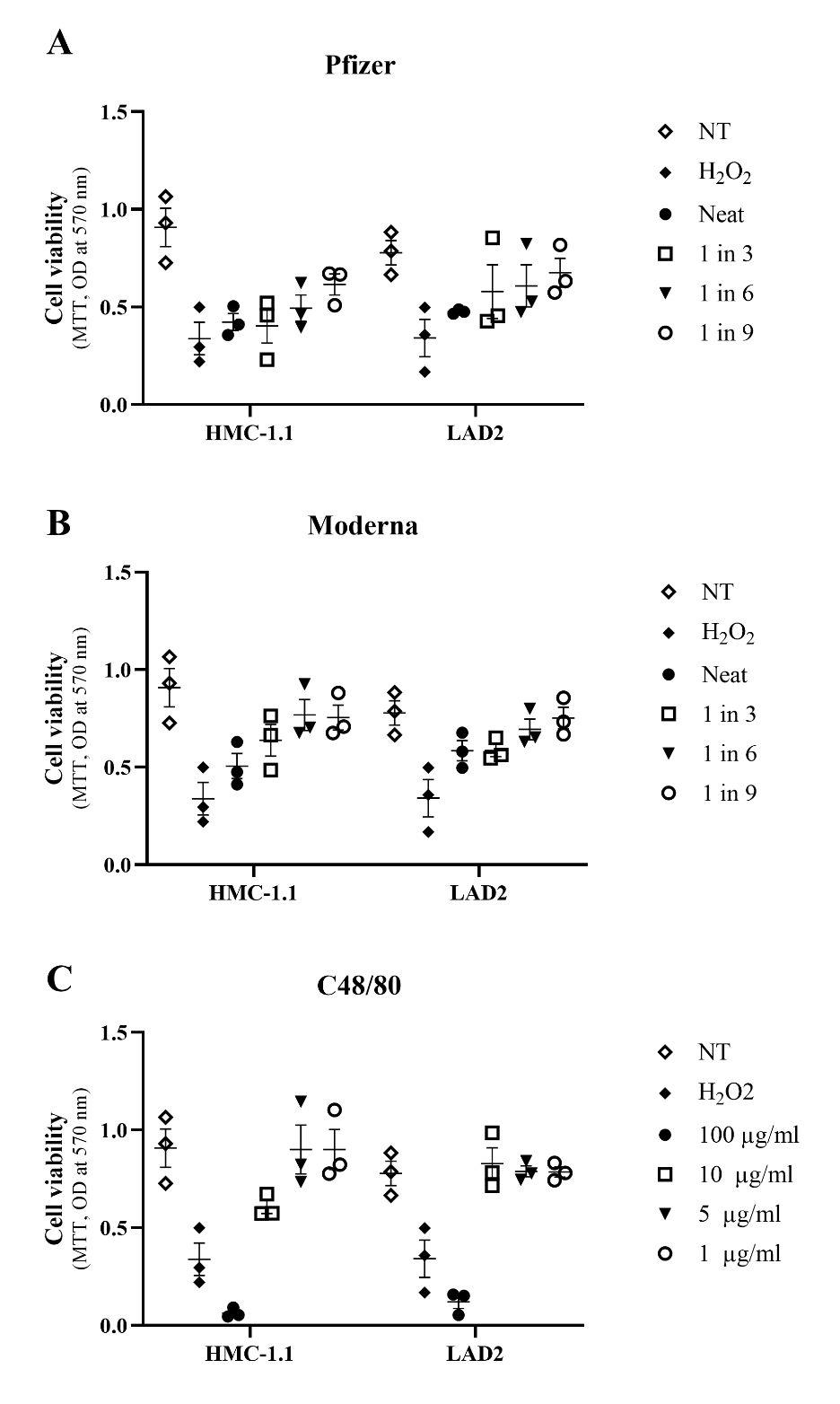


**Supplementary Figure S12: Cell viability data using the MTT assay after mast cell line challenge with two mRNA vaccines**

Cell viability after incubation with Comirnaty (BNT162b2, BioNtech/Pfizer), H_2_O_2_ or buffer (A); Spikevax (mRNA-1273, Moderna), H_2_O_2_ or buffer (B); C48/80 (mast cell degranulating factor), H_2_O_2_ or buffer (C). Subsequently cell viability was determined using the MTT assay. Each point represents one individual experiment with averaged technical duplicates. Each substance was tested on three different days. Results are presented as individual measurements with their respective mean (± SEM). NT = No treatment; C48/80 = Compound 48/80; OD = optical density; neat = no dilution.

**Supplementary References**

1. Butterfield JH, Weiler D, Dewald G, Gleich GJ. Establishment of an immature mast cell line from a patient with mast cell leukemia. Leuk Res. 1. Januar 1988;12(4):345–55.

2. Kirshenbaum AS, Akin C, Wu Y, Rottem M, Goff JP, Beaven MA, u. a. Characterization of novel stem cell factor responsive human mast cell lines LAD 1 and 2 established from a patient with mast cell sarcoma/leukemia; activation following aggregation of FcεRI or FcγRI. Leuk Res. 1. August 2003;27(8):677–82.

3. Hermans MAW, Schrijver B, van Holten-Neelen CCPA, Gerth van Wijk R, van Hagen PM, van Daele PLA, u. a. The JAK1/JAK2- inhibitor ruxolitinib inhibits mast cell degranulation and cytokine release. Clin Exp Allergy. 2018;48(11):1412–20.

4. Hermans MAW, van Stigt AC, van de Meerendonk S, Schrijver B, van Daele PLA, van Hagen PM, u. a. Human Mast Cell Line HMC1 Expresses Functional Mas-Related G-Protein Coupled Receptor 2. Front Immunol [Internet]. 2021 [zitiert 22. April 2022];12. Verfügbar unter: https://www.frontiersin.org/article/10.3389/fimmu.2021.625284

5. Kuehn HS, Radinger M, Gilfillan AM. Measuring Mast Cell Mediator Release. Curr Protoc Immunol. 2010;91(1):7.38.1-7.38.9.

6. Boehm T, Ristl R, Joseph S, Petroczi K, Klavins K, Valent P, u. a. Metabolome and lipidome derangements during a severe mast cell activation event in a patient with indolent systemic mastocytosis. J Allergy Clin Immunol. 14. April 2021;148(6):1533–44.

7. Boehm T, Ristl R, Mühlbacher J, Valent P, Wahrmann M, Jilma B. Massive release of TH2 cytokines induced a cytokine storm during a severe mast cell activation event in a patient with indolent systemic mastocytosis. J Allergy Clin Immunol. 1. August 2022;150(2):406-414.e16.

8. Theoharides TC, Tsilioni I, Ren H. Recent advances in our understanding of mast cell activation – or should it be mast cell mediator disorders? Expert Rev Clin Immunol. Juni 2019;15(6):639.
